# Development and characterization of agonistic antibodies targeting the Ig-like 1 domain of MuSK

**DOI:** 10.1038/s41598-023-32641-1

**Published:** 2023-05-08

**Authors:** Jamie L. Lim, Roy Augustinus, Jaap J. Plomp, Kasra Roya-Kouchaki, Dana L. E. Vergoossen, Yvonne Fillié-Grijpma, Josephine Struijk, Rachel Thomas, Daniela Salvatori, Christophe Steyaert, Christophe Blanchetot, Roeland Vanhauwaert, Karen Silence, Silvère M. van der Maarel, Jan J. Verschuuren, Maartje G. Huijbers

**Affiliations:** 1 Department of Human Genetics, Leiden University Medical Center, Einthovenweg 20, 2300 RC Leiden, The Netherlands; 2 Department of Neurology, Leiden University Medical Center, Leiden, The Netherlands; 3 Department PDC-Pathologie, Leiden University Medical Center, Leiden, The Netherlands; 4 Veterinary Faculty, Department Clinical Sciences, Universiteit Utrecht, Utrecht, The Netherlands; 5 argenx BV, Zwijnaarde, Belgium

**Keywords:** Molecular medicine, Neuromuscular disease

## Abstract

Muscle-specific kinase (MuSK) is crucial for acetylcholine receptor (AChR) clustering and thereby neuromuscular junction (NMJ) function. NMJ dysfunction is a hallmark of several neuromuscular diseases, including MuSK myasthenia gravis. Aiming to restore NMJ function, we generated several agonist monoclonal antibodies targeting the MuSK Ig-like 1 domain. These activated MuSK and induced AChR clustering in cultured myotubes. The most potent agonists partially rescued myasthenic effects of MuSK myasthenia gravis patient IgG autoantibodies in vitro. In an IgG4 passive transfer MuSK myasthenia model in NOD/SCID mice, MuSK agonists caused accelerated weight loss and no rescue of myasthenic features. The MuSK Ig-like 1 domain agonists unexpectedly caused sudden death in a large proportion of male C57BL/6 mice (but not female or NOD/SCID mice), likely caused by a urologic syndrome. In conclusion, these agonists rescued pathogenic effects in myasthenia models in vitro*,* but not in vivo. The sudden death in male mice of one of the tested mouse strains revealed an unexpected and unexplained role for MuSK outside skeletal muscle, thereby hampering further (pre-) clinical development of these clones. Future research should investigate whether other Ig-like 1 domain MuSK antibodies, binding different epitopes, do hold a safe therapeutic promise.

## Introduction

Impairment of transmission at the neuromuscular junction (NMJ) is a hallmark or component of a range of neuromuscular disorders (NMDs) and motor neuron diseases (MNDs), including myasthenia gravis (MG), amyotrophic lateral sclerosis (ALS), spinal muscular atrophy (SMA) and Duchenne muscular dystrophy (DMD)^1,2^. The prevalences of most NMDs range between 1 and 10 per 100,000^3^. There is a large unmet medical need among these patients, as there are no cures available. Theoretically, these disorders would benefit from improving neuromuscular transmission and NMJ stability as this may (partially) delay onset, slow down progression, halt or even rescue muscle weakness in these patients^4,5^. In healthy developing and mature NMJs, synaptic stability is tightly orchestrated by the agrin-low-density LDL receptor-related protein 4 (Lrp4)-muscle-specific kinase (MuSK)-downstream of kinase 7 (Dok-7) trophic signalling pathway. This pathway starts at the motor neuron releasing agrin that binds Lrp4 and thereby facilitates the interaction between Lrp4 and MuSK. In response, MuSK dimerizes and becomes an active kinase with the help of intracellular Dok-7^6^. The MuSK kinase activity triggers multiple (partly unknown) intracellular signalling pathways of which one results in clustering of acetylcholine receptors (AChRs). Aside from this anterograde signalling, this pathway is also thought to facilitate retrograde signalling by Lrp4 to establish motor nerve terminals at prepatterned muscle areas^7^. Absence of any of the proteins involved in this signalling pathway is incompatible with life as NMJs fail to form, and skeletal muscles, including respiratory muscles, do not function^8–10^. Perturbation of the function of these proteins during life, e.g. by IgG4 autoantibodies in MuSK MG, results in (potentially severe) muscle weakness^11^.

Stimulation of the agrin-Lrp4-MuSK-Dok7 pathway, e.g. by Dok7 gene therapy or an engineered agrin protein, has a therapeutic effect in animal models for AChR MG^12^, congenital myasthenic syndrome (CMS)^13^, ALS^14^, SMA^15,16^ and several forms of muscular dystrophies^17^. Overexpression of MuSK in a mouse model for ALS^18^ resulted in delayed disease onset, reduced muscle denervation and improved motor function, whilst overexpression of Lrp4 in skeletal muscle partially rescued NMJs in the *mdx* mouse model for DMD^19^. Moreover, MuSK kinase activity can be stimulated with agonistic antibodies or antibody fragments targeting the MuSK Frizzled (Fz) domain: monoclonal antibody (mAb)13 improved survival and motor performance in an animal model for ALS^20,21^ and reduced NMJ denervation in the SMA delta 7 mouse model^22^, whereas mAb X17 rescued synapse formation, motor function and lethality in a Dok7 CMS mouse model^23^. Overall, these studies demonstrate that targeting the MuSK signalling pathway can be therapeutic, also in NMDs in which MuSK perturbation is not necessarily the main cause of disease.

Recently, we derived several monoclonal antibodies from MuSK MG patients which engage with high affinity the Ig-like 1 domain of MuSK^24,25^. These studies revealed that the functional monovalency of IgG4 MuSK MG antibodies is crucial for inducing myasthenia. The bivalent forms of these MuSK antibodies behave as (partial) MuSK agonists, i.e. they induce MuSK kinase activity, AChR clustering and, depending on the clone, cause no or a milder myasthenic phenotype in mice. We hypothesized that bivalent Ig-like 1 domain MuSK antibodies could thus form potential therapeutics for NMDs with impaired NMJ function by stimulating the MuSK signalling cascade. Therefore, we developed a range of antibodies binding the N-terminal Ig-like 1 domain of MuSK and investigated their agonistic properties and therapeutic potential.

## Materials and methods

### Antibody isolation, production and selection

Two types of monoclonal MuSK Ig-like 1 domain antibodies were used in this study. The first set was isolated from a MuSK MG patient as described previously^24^. In short, patients with MuSK MG were recruited in our MG outpatient clinic at the Leiden University Medical Centre (LUMC) and were selected based on the presence of a positive MuSK antibody test (RSR Ltd). The study was conducted in accordance with the Declaration of Helsinki and was approved by the local medical ethics committee (The Medical Ethics Committee Leiden The Hague Delft) and patients signed informed consent. To avoid immunogenicity in future preclinical development, the antibodies were germlined and derisked (effector functions knocked down and sequence optimized)^26^, resulting in 20 variants of the original five clones. An overview of the clones, the variants introduced and their functional characteristics is given in Supplementary Table S1. The second set of antibodies resulted from libraries generated from two llamas that were immunized with recombinant human MuSK-Fc chimera protein (R&D Systems, cat. 9810-MK). RNA from peripheral blood lymphocytes was reverse transcribed to cDNA and the Fabs then cloned into a phagemid vector as described in de Haard et al.^27^ and used for phage display selection against recombinant different domains of MuSK to isolate anti-domain MuSK specific Fabs (SIMPLE antibody™ platform of argenx). The variable domains of both types of antibodies (patient and llama-derived) were reformatted as a full human immunoglobulin G1 (IgG1) equipped with the Leu234Ala/Leu235Ala (LALA) mutations to knock down the antibody effector functions^26^ and deletion of the C-terminal lysine (delK) to avoid heterogeneity. All antibodies were formulated in PBS with 0.02% Tween-80. Endotoxin levels were determined at Eurofins (cut-off value of < 0.1 EU/mL). The llama-derived antibodies were further selected for their binding features on mouse and human recombinant MuSK. All antibodies were produced by transient transfection in HEK293-E 253 cells (obtained from ImmunoPrecise Antibodies) and purified by MabSelect SuRe™ LX beads and size exclusion chromatography to remove potential aggregate of antibodies.

Patient polyclonal IgG(4) was derived from MuSK MG patients undergoing therapeutic plasmapheresis. For the use of the plasmapheresis material, patients signed informed consent (no permission was required from an ethical committee for the use of this “waste” material). The different plasmapheresis batches per patient were transported to Immunoprecise Antibodies Ltd, pooled and purified for IgG4 using a 35 mL CaptureSelect-hIgG4 XK26/20 column (Pharmacia Biotech). The concentrated IgG4 was shipped back to the LUMC for storage at − 20 °C and further use. The patient plasmapheresis batches that were selected for IgG4 purification had highly MuSK reactive IgG4 (tested in a MuSK enzyme-linked immunosorbent assay [ELISA], data not shown) and were of a large volume. For polyclonal patient total IgGs, plasmapheresis material was purified on a 200 mL CaptureSelect IgG total XK20/50 column (Pharmacia Biotech) at the LUMC and stored at − 20 °C for further use.

### Affinity measurements

Anti-human Fc antibody (Jackson ImmunoResearch, cat. 109-005-098) was diluted in 10 mM sodium acetate pH 4.5 (Cytiva cat. BR100350) and immobilized on flow channels Fc1 and Fc2 on a CM5 chip series S (Cytiva cat. 29149603) using amine coupling chemistry (amine coupling kit Cytiva, cat. BR100050) on a Biacore™ T200 SPR device (Cytiva cat. 28975001). HBS-EP + pH 7.4 (diluted in MilliQ H_2_O from a 10 × stock HBS-EP + pH 7.4 [Cytiva, BR100826]) was sterile-filtered and used as running buffer at a flow rate of 30 µL/min. Antibodies of interest were captured at around 150 RU in flow channel Fc2 with a contact time of 30 s at pH 7.4 and a flow rate of 10 µL/min, followed by a stabilization time of 30 s. Next, the antigen was injected as a 3 point 1 over 4 dilution series over flow channels Fc1 and 2 starting from 10 mM applying an association time of 5 min, a dissociation time of 10 min and a flow rate of 30 µL/min. Regeneration was done by injection of 10 mM glycine–HCl pH 1.5 (Cytiva cat. BR100354). Double referenced sensorgrams were fitted using a 1:1 binding model to obtain the on-rate ka (1/Ms), off-rate kd (1/s) and the affinity KD (M).

### Epitope mapping

The respective human Fabs of the monoclonal antibodies of interest were produced in HEK293 cells (obtained from ImmunoPrecise Antibodies) and were coated on a Microlon half-area ELISA plate (Greiner cat. 675061) diluted at a concentration of 1 µg/mL in 1 × PBS and incubated overnight at 4 °C. The next day the ELISA plate was washed 3 times with 1 × PBS-0.05% Tween20 pH 7.4 and blocked with 100 µL/well 1% casein in 1 × PBS, followed by an incubation of 1 h on an ELISA plate shaking platform at room temperature. Recombinant Human MuSK His-tag Protein (R&D Systems, 10189-MK-MTO) diluted to 1 µg/mL in 1 × PBS 0.1% casein was added to each well and was incubated 1 h at room temperature on an ELISA shaking platform. After washing each well with 1 × PBS-0.05% Tween20 pH 7.4 mAbs of interest were diluted in 1 × PBS 0.1% casein at a concentration of 1 µg/mL for 1 h at room temperature while shaking. After 1 h the plates were washed with 1 × PBS-0.05% Tween20 pH 7.4 and detection antibody/well was added (Peroxidase affinipure goat anti-human IgG Fc fragment specific, Jackson ImmunoResearch 109-035-098, diluted 1 over 5000 in 1 × PBS 0.1% casein) and was incubated at room temperature while shaking. After 1 h the plates were washed with 1 × PBS-0.05% Tween20 pH 7.4 and TMB (Merck cat. CL07-1000ML) was added and color development was monitored. Reaction was terminated by adding 0.5N H_2_SO_4_ (ChemLab cat. CL052615) and OD was measured at 450 nm (reference 620 nm).

### C2C12 culturing

C2C12 mouse myoblast cells (Cell Lines Service) were cultured in DMEM GlutaMAX (31966 Gibco) with added 10% fetal bovine serum and 1% penicillin/streptomycin (proliferation medium) until 60–70% confluency. For differentiation into myotubes the cells were plated on 10 cm dishes (Greiner Bio-One, 664160, 58 cm^2^, MuSK phosphorylation assay) or on 96-wells plates (Greiner µclear, 655090, 0.32 cm^2^/well, AChR clustering assay), at 1.25 × 10^4^ cells/cm^2^ in proliferation medium. Myoblast fusion into myotubes was induced after reaching 90–95% confluency (~ 2 days), by culturing in differentiation medium consisting of 2% horse serum, 1% penicillin/streptomycin and in DMEM GlutaMAX™. Medium was changed every 2 days. After 5 days the myoblasts were fused into myotubes. Cells were kept at 37 °C with 5% CO_2_ during culturing.

### MuSK tyrosine phosphorylation assay

C2C12 myotubes on 10 cm dishes were stimulated in different experimental conditions using 10 ng/mL agrin (Rat c-term agrin 3,4,8 R&D550AG) and/or 1.1 µg/mL mAbs (mono or bispecific) and/or 2.25 mg/mL patient-purified IgG-total (Patient (Pt)#1) for 30 min at 37 °C, unless otherwise specified. Due to a batch concentration problem, 13-3D10wt was not included in the MuSK tyrosine phosphorylation (MuSK-P) assay. To obtain quantitative MuSK-P data, myotubes were extracted in lysis buffer (30 mM triethanolamine, 1% NP-40, 50 mM NaF, 2 mM Na-orthovanadate, 1 mM Na-tetrathionate, 5 mM EDTA, 5 mM EGTA, 1 mM N-ethylmaleimide, 50 mM NaCl, 1 × protease inhibitor cocktail, 1 × phosphatase inhibitor cocktail) followed by centrifugation (5000 g). To precipitate MuSK, the lysate normalized for protein content was incubated using 5 µL/sample rabbit anti-MuSK polyclonal serum (ab94276 or ab94277, a kind gift by Prof. Steve Burden, NYU Medical School) or using 1 µg/sample 11-3F6 IgG1 anti-MuSK antibody at 4 °C overnight. Using protein A agarose beads (11134515001, Roche), the bound antigen–antibody complexes were captured. After 5 min incubation at 95 °C with sample buffer containing reducing agent dithiothreitol (DTT), the bead-precipitated proteins were run on SDS-PAGE gel electrophoresis and transferred to a polyvinylidene difluoride membrane. Goat anti-rat MuSK (R&D AF 562) and mouse anti-pTyr clone 4G10 (Millipore 05-321) were used as primary antibodies for the detection of MuSK protein and phosphorylated MuSK. As secondary antibodies for the fluorescent signal donkey anti-mouse-680RD (926-68072, Licor) and donkey anti-goat-800CW (926-32214, Licor) were used. Fluorescence intensity of bound antibodies was detected using the Odyssey CLx (Licor). Tyrosine phosphorylation experiments were repeated three times and normalized for the effect of agrin, unless otherwise stated.

### AChR clustering assay

AChR clustering in myotubes was induced for 24 h with varying experimental conditions using 10 ng/mL agrin and/or 1.1 µg/mL mAbs (mono or bispecific) and/or 2.25 mg/mL Pt#1 MuSK-reactive total IgG. In therapeutic experiments, the inhibiting bispecific IgG4 or patient IgG was incubated for 30 min before adding the agonist antibody and finalizing the remaining 23.5 h incubation. The experimental investigator was blinded for the conditions. Twenty-four hour incubation was followed by incubation with Alexa Fluor 488-conjugated α-bungarotoxin (B13422, Thermo Fisher) to stain AChRs and Hoechst 33342 (H1399, Thermo Fisher) to stain nuclei, for 30 min at 37 °C. The cells were fixed in 4% w/v paraformaldehyde for 10 min at room temperature and then used for imaging. Microscopy was performed with 20 × magnification on a Leica AF6000 fluorescence microscope. Four visual fields per well and five wells per condition were selected, based on evenly spread and clearly visible mature myotubes in the brightfield channel. Analysis of AChR-positive cluster count and area size were performed using ImageJ (1.52n) and MATLAB (R2018b). The number of mature clusters (≥ 15 µm^2^) per condition and its distribution of all cluster sizes were quantified. Only mature clusters were included for the cluster count analysis, as they represent the most functionally relevant clusters^28–30^. AChR clustering distribution graphs were produced by deriving a fitted line from a histogram containing all quantified cluster area sizes in MATLAB. AChR clustering experiments were repeated 3 times and normalized for the effect of agrin, unless otherwise stated.

### Passive transfer studies in mice

#### Mice

The five different passive transfer tolerability or MuSK MG mouse studies described in this paper were conducted at three different independent institutions. For clarity, an overview of the study design and outcomes can be found in Supplementary Table S2 and Supplementary Figure S1. Mouse studies in C57BL/6J mice were conducted at the laboratory of Christopher Borg (Besançon, France) (Supplementary Fig. S1a), at the Jackson (JAX) Laboratory (Bar Harbor, ME, USA) (Supplementary Fig. S1b) and at the Leiden University Medical Center (LUMC; Leiden, The Netherlands) (Supplementary Fig. S1c). The tolerability (Supplementary Fig. S1d) and MuSK MG (Supplementary Fig. S1e) studies in NOD/SCID mice were also conducted at the LUMC. C57BL/6JIco mice used in the LUMC study were purchased from Charles River (Wilmington, MA, USA) and those used in the Besançon study from Charles River (Domaine des Oncins, L’arbresle Cedex, France). Mice used in the JAX study were bred locally at the Jackson Laboratory. Original breeders of immunodeficient NOD.CB17-Prkdcscid/J (NOD/SCID) mice were also purchased from Jackson laboratory. All mice were approximately two months old at the start of the studies. Sterilized food and drinking water were provided ad libitum. NOD/SCID mice were housed in sterile individually ventilated cages. All experiments were carried out according to Dutch law and Leiden University guidelines, including approval by the local Animal Experiments Committee (Instantie voor Dierenwelzijn). The mouse experiments at the Besançon and Jackson Laboratory were performed based on French (The Animal Experimentation Ethics Committee) and American (Institutional Animal Care and Use Committee) law and ethics guidelines.

#### Dosing and assessments

To test the tolerability of the Ig-like 1 candidate antibodies in mice, we used 25 NOD/SCID mice at the LUMC. After measuring body weight and establishing baseline values for the in vivo neuromuscular tests during 6 days, the mice were injected intraperitoneally every 3 days with 20 mg/kg of Ig-like 1 binding MuSK agonist antibodies 13-3D10a, 13-4D3a, 11-3F6c or 11-3D9b (all antibodies generated contained the same IgG1-LALA-delK backbone), or were left untreated for 25 days. As positive control we included a monospecific version of the Ig-like 1 binding antibody 13-3B5 (produced with a hIgG4S228P Fc tail in CHO cells), known to be able to induce myasthenia in NOD/SCID mice^25^. Each treatment group consisted of 2 male and 2 female mice, except for the untreated group which included 2 male and 3 female mice. The clinical signs test consisted of daily measurements of body weight and neuromuscular performance in grip strength and hanging time on an inverted mesh (maximum 180 s, 3 attempts)^11^.

At Besançon, a total of 36 C57BL/6 mice (18 male, 18 female) were injected intraperitoneally every 3 days for 8 weeks in a blinded fashion with 20 mg/kg or 5 mg/kg of antibodies 13-4D3, 11-3F6c, or 11-3D9b or 20 mg/kg of 13-3D10a, MuSK Fz domain binding antibody mAb13 (benchmark, Genentech, known MuSK agonistic antibody, positive control) or Mota (RSV binding antibody, irrelevant binding domain, negative isotype control) in the hIgG1-LALA-delK backbone (C-terminal lysine was removed genetically to avoid heterogeneity). Each treatment group included 2 male and 2 female mice.

At the Jackson laboratory, C57BL/6 mice (N = 20) received intraperitoneal injections with 0.03, 0.1, 0.3, 1 or 5 mg/kg 11-3F6c or Mota (2 males, 2 females per treatment group) every 3 days for 5 weeks.

At the LUMC, a total of 24 C57BL/6 mice (12 males, 12 females) were injected every 3 days in an investigator-blinded fashion with either 11-3F6c (n = 18), or Mota (n = 6). Six mice (3 males and 3 females) were euthanized after 5, 8 and 11 weeks of 11-3F6c injections consecutively, whilst 6 Mota mice were euthanized after 12 weeks of injections, following endpoint analyses.

For passive transfer of patient purified IgG4 in the MuSK MG mouse model, the minimal dose inducing a maximal myasthenic phenotype (40 mg/kg patient 2017-004 polyclonal IgG4) was administered to 2-month-old female NOD/SCID mice daily. One day prior to injection of IgG4, mice were injected every 3 days with either 2.5 mg/kg 11-3F6c (n = 6), or Mota (n = 6) in a blinded fashion. The daily IgG4 dose was dissolved in either sterile PBS with Mota or sterile PBS with 11-3F6c on the days of planned agonist administration. Before the start of injections, (daily) body weight and baseline values for neuromuscular performance were established of each mouse, and continued until humane endpoint. If body weight loss of ≥ 20% occurred, compared with the starting weight, mice were euthanized by CO_2_ inhalation (i.e. humane endpoint). Blood samples for analyzing serum titers were taken through tail vein puncture shortly before each injection of 11-3F6c (and IgG4).

#### Repetitive nerve stimulation electromyography

Repetitive nerve stimulation electromyography (RNS-EMG) was conducted at the LUMC on C57BL/6 and NOD/SCID mice from the Besançon and LUMC studies as described. Mice were anaesthetized with a 1.5:1 (v/v) mixture of ketamine hydrochloride (Nimatek; 100 mg/mL, Eurovet) and medetomidine hydrochloride (Domitor; 1 mg/mL, Pfizer), at 1.25 μL/g mouse body weight, adjusted with Ringer solution to 200 μL volume and administered intraperitoneally. Mice were maintained at 37 °C on a heating pad. A grounding needle electrode was inserted subcutaneously in the right thigh. Stimulation needle electrodes were inserted near the sciatic nerve in the left leg thigh. Subcutaneous recording needle electrodes were inserted near the calf muscles of the left leg. Grounding and recording electrodes were coupled via an AI402 pre-amplifier to a Cyberamp-380 signal conditioner (Axon Instruments/Molecular Devices). The nerve was stimulated supramaximally from a computer-controlled programmable electrical stimulator (AMPI). Trains of 20 stimuli were applied at increasing frequencies of 40 Hz, with a 30 s pause between trains. Compound muscle action potentials (CMAPs) were digitized using a Digidata 1440 interface and Axoscope 10 (Axon Instruments/Molecular Devices). Peak–peak amplitudes were determined in Clampfit 11 (Axon Instruments/Molecular Devices). After completing the recordings, mice were euthanized by CO_2_ inhalation without recovery from anesthesia and muscles were dissected for the studies described below.

C57BL/6 mice from the JAX study were analyzed for neuromuscular transmission using RNS-EMG at the Jackson laboratory using a similar method as described above, except mice were anesthetized with isoflurane for up to 5 min with 2–3% isoflurane in O_2_. In addition, for RNS, stimulation intensity was increased to 120% of the intensity required to record the maximum CMAP. One train of 20 stimuli was administered at 40 Hz, while the CMAPs were recorded. Decrement upon RNS was expressed as the percentage of decrease of the CMAP from the 1st stimulus of each train.

#### Ex vivo muscle contraction studies

Muscle contraction was analyzed in dissected muscles from NOD/SCID and C57BL/6 mice (from the LUMC studies only). Contraction force of left phrenic nerve-hemidiaphragms was recorded in Ringer’s medium containing (in mM): NaCl 116, KCl 4.5, CaCl_2_ 2, MgCl_2_, NaH_2_PO_4_, NaHCO_3_, glucose, pH 7.4) at room temperature (20–22 °C) with a force transducer (type K30, Harvard Apparatus, Hugo Sachs Elektronik GmbH), connected to an amplifier TAM-A 705/1 (Hugo Sachs Elektronik). The signal was digitized using a Digidata 1440 digitizer (Axon Instruments/Molecular Devices), connected to a PC running Axoscope 10 (Axon Instruments). The phrenic nerve was stimulated supramaximally once every 5 min with 280 stimuli of 100 µs duration at 40 Hz, i.e. for 7 s. The safety factor of neuromuscular transmission was assessed by measuring contraction force before and after equilibration with 125 nM d-tubocurarine (Sigma-Aldrich). The area under each contraction curve was determined in later off-line analyses, using Clampfit 11 (Axon Instruments).

#### Fluorescence microscopy of NMJs

Morphology of NMJs was analyzed in diaphragms of all surviving mice in all studies and in the epitrochleoanconeus (ETA) muscle for the two LUMC studies. Diaphragm strips (right hemidiaphragm, most dorsal area) and ETA muscles were fixed in 1% paraformaldehyde in PBS for 1 h, washed in PBS and incubated for 2.5 h with 1 µg/mL Alexa Fluor 488 conjugated α-bungarotoxin to stain AChRs, followed by PBS wash (1 h), all at room temperature. Muscles were whole-mounted and viewed under an epi-fluorescent microscope (Zeiss Axioskop), using identical settings for all muscles. Low-magnification overview pictures (5 × objective), containing about 150 NMJs, were taken using Axiovision software (Zeiss). The integrity of the AChR area at NMJs was semi-quantitively scored by the investigator (blinded to the treatment) either as ‘normal’ (i.e. a clear, continuous pretzel-like structure without much fragmentation of AChR clustering) or ‘abnormal’ (i.e. fragmented and/or faint AChR clusters). The proportion of abnormal NMJs was calculated by dividing the number of NMJs scored as abnormal by the total number of NMJs evaluated in the picture.

### Statistical tests

One-way-ANOVA with Dunnett’s multiple comparison test, (multiple) unpaired t-tests with Holm-Šídák or Bonferroni corrections for multiple testing, or a Log-rank (Mantel-Cox test) were performed wherever appropriate. Differences with P values < 0.05 were considered statistically significant. GraphPad Prism 9.3.1 was used for statistical analyses and to test data sets for normality.

The studies reported in this paper were conducted according to the ARRIVE guidelines (https://arriveguidelines.org).

## Results

### Antibody selection and characterisation

**Figure 1:**
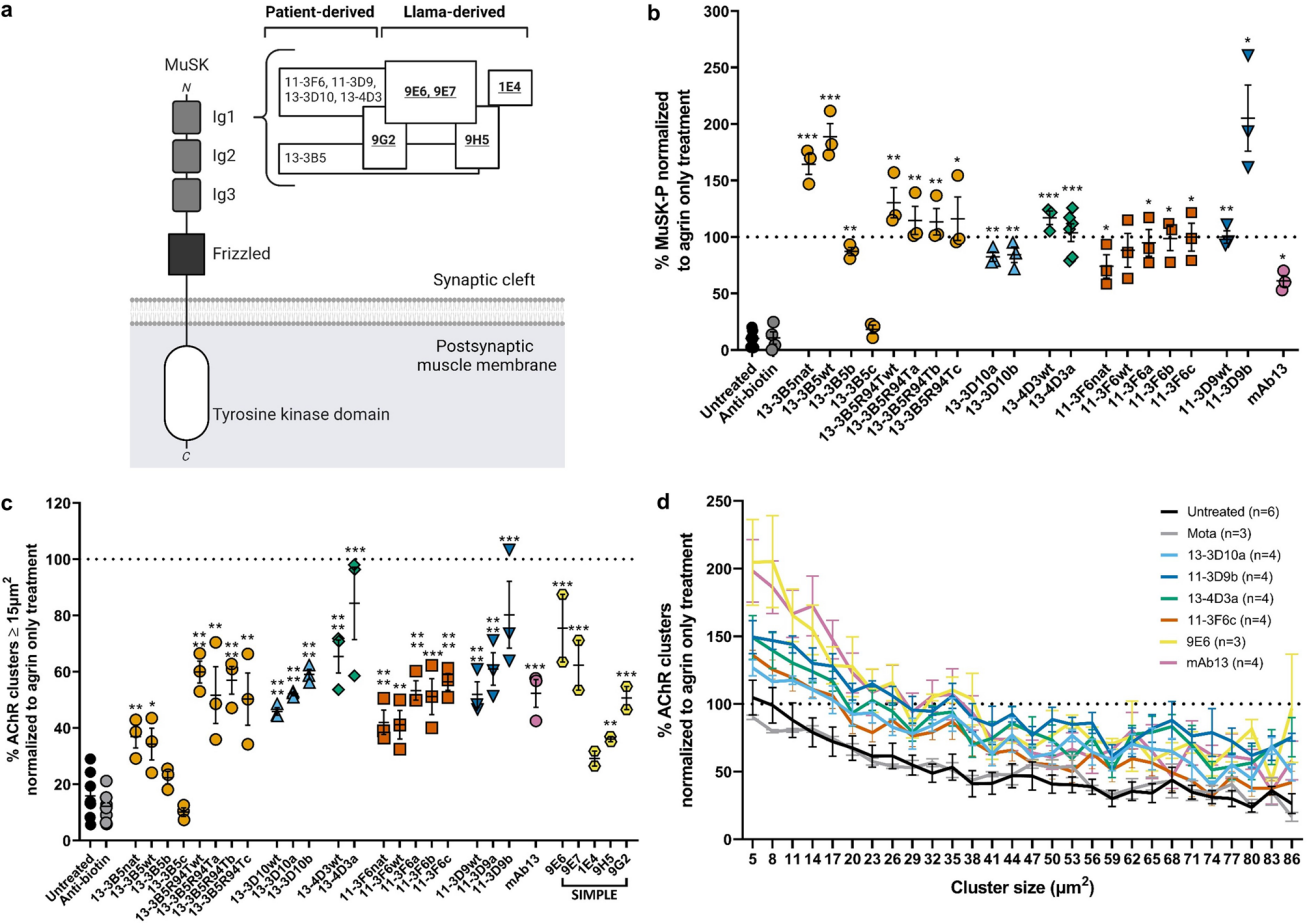
Ig-like 1 binding MuSK antibodies have varying agonist potential in vitro. (**a**) Graphic depiction of the epitopes of the Ig-like 1 agonist antibodies on MuSK. Antibodies in the same box share an epitope. Overlapping boxes represent partial sharing of an epitope. Bold-underscored clones represent the antibodies produced in llamas. (**b**) MuSK phosphorylation levels induced by MuSK agonist antibodies as expressed by percentage normalized to agrin only treatment. Colors identify groups of antibody clones derived from the same parental clone. (**c**) Percentage of AChR clusters induced by MuSK agonist antibodies, with a size larger than 15 µm^2^, normalized to agrin only treatment. (**d**) Percentage of different AChR cluster sizes induced by the different candidate antibodies normalized to the agrin only condition. Data information: for statistical analysis in b and c, an unpaired t-test with Bonferroni correction was performed to compare the different agonists versus biotin control. Statistical significance was set at *P < 0.05; **P < 0.01; ***P < 0.001; ****P < 0.0001.

From the MuSK MG patient-derived parental clones 13-3B5, 11-3F6, 13-3D10, 11-3D9 and 13-4D3^24^, 15 additional humanized and derisked variants were produced. In addition, 5 MuSK llama-derived antibodies were generated (9E6, 9E7, IE4, 9H5, 9G2). Affinity to MuSK, species cross reactivity, thermotolerance and detailed epitope mapping was investigated for all 25 antibodies. Epitope mapping using a competition ELISA and SPR Biacore™ revealed that one dominant epitope could be identified within the Ig-like 1 domain of the patient-derived antibodies (Fig. 1a).

Eight antibodies (of patient and llama origin) binding to the Ig-like 1 domain had (partially) overlapping epitopes. Furthermore, at least one other epitope could be identified for which the antibodies could not compete (13-3B5). Germlining and derisking these clones largely did not affect their binding features except for the R94T variant in 13-3B5, which resulted in significant loss of MuSK binding and activating capacity (see further below). The patient-derived clones were fully cross-reactive with MuSK protein from mice and humans. Llama-derived clones showed good cross-reactivity with human MuSK, but showed significantly lower affinity for mouse MuSK (data not shown). A summary of the characteristics of the key antibodies can be found in Supplementary Table S1.

### MuSK agonist antibodies show varying agonistic potential on MuSK activation and AChR clustering in mouse myotube cultures

C2C12 myotube cultures harbour all the necessary cellular machinery for formation of postsynaptic NMJ structures except for the presence of neuronal agrin. Addition of agrin induces AChR patches reminiscent of early NMJs. To investigate the agonistic potential of the selected MuSK antibodies, their effect on MuSK-P and AChR clustering were tested compared to agrin (the natural MuSK ligand), mAb13 (MuSK Fz-domain binder, positive control), and a negative control biotin or isotype control Mota antibody. All MuSK agonist antibodies (both patient-derived and llama-derived), except for 13-3B5c, were able to induce MuSK-P and AChR clustering to a varying degree, independent from agrin (Fig. 1b,c). The introduction of the R94T mutations in 13-3B5 variants critically impaired the agonistic potential of the 13-3B5c clone. 11-3D9b and 13-3B5wt were most potent in inducing MuSK-P, while 13-4D3a and 11-3D9b were most potent in inducing AChR clustering. On average there was a moderate and significant correlation between agonistic potential on MuSK-P and AChR clustering (R = 0.59, P = 0.0029) (Supplementary Fig. S2a). The majority of antibodies induced clustering levels of around 50% to that of agrin. Surprisingly, all humanized variants induced more clusters than their parental clones (named “nat” or “wt”), except for 13-3B5b and 13-3B5c.

To investigate whether the antibodies induce AChR clusters of similar sizes and thus maturity, we generated cluster distribution graphs (Fig. 1d). All antibodies produced more smaller-sized clusters (< 15 µm^2^), but less mature clusters (≥ 15 µm^2^), compared to agrin. Co-incubation of myotubes with MuSK agonists (mAb13, Supplementary Fig. S2b; parental clones 13-3B5 IgG4S228P and 11-3F6 IgG4S228P^25^) and agrin resulted in cluster counts (≥ 3 µm^2^ and ≥ 15 µm^2^) approximately equal or less to the antibody-only levels, thus a synergistic effect was not observed. Importantly, antibody EC50-value negatively correlated with the potential to induce AChR clustering (R =  − 0.66, P = 0.0049) confirming that binding strength (i.e. lower EC50 values) potentiates MuSK antibody agonism (Supplementary Fig. S2c). Given the large differences in agonistic potential of these MuSK antibodies, we prioritized the most potent inducers of AChR clustering (11-3F6c, 13-3D10a, 11-3D9b, 13-4D3a and 9E6) for further experiments.

### MuSK agonist antibodies can rescue MuSK myasthenic-like effects in vitro

**Figure 2:**
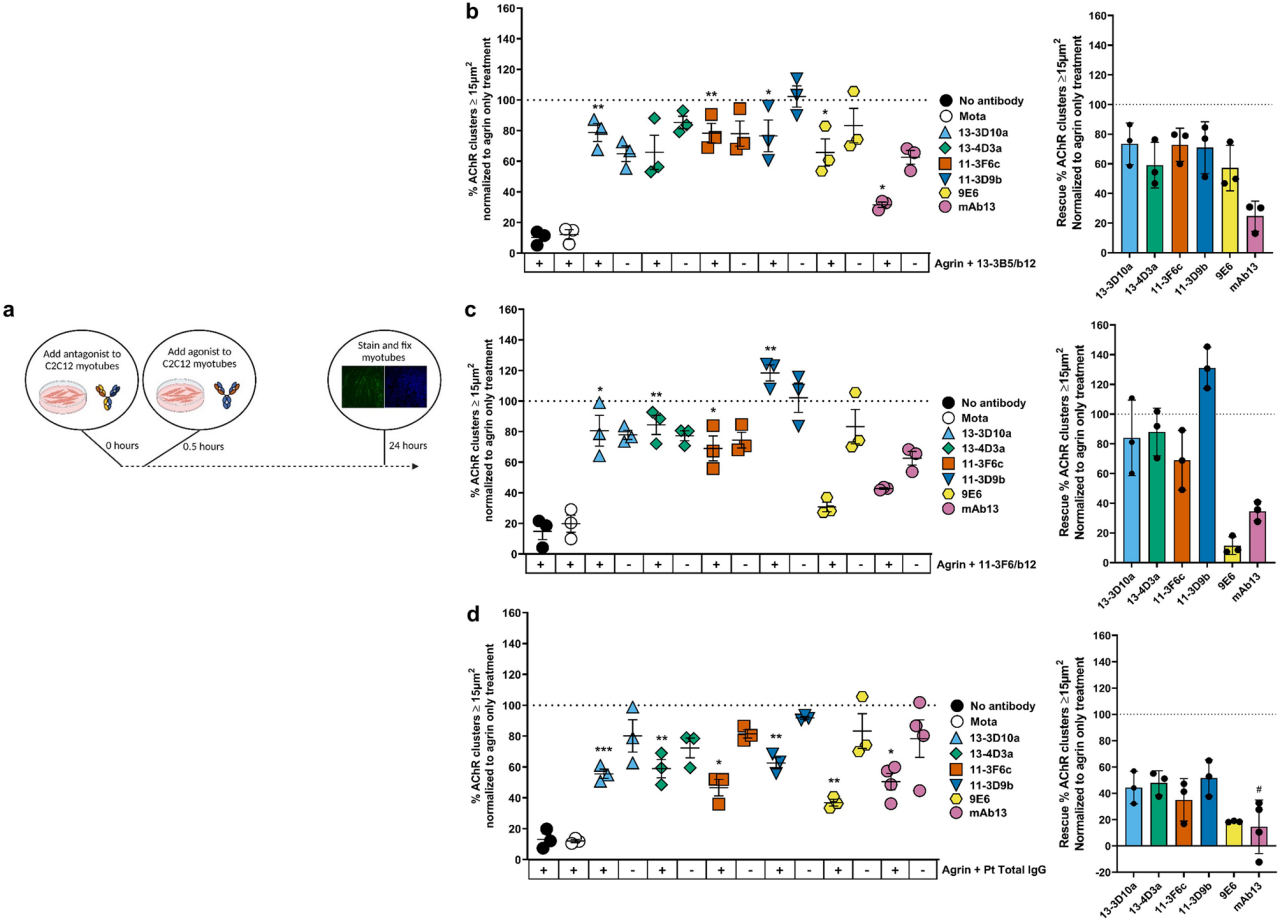
MuSK agonist antibodies (partially) rescue AChR declustering induced by monovalent monoclonal MuSK antibodies or polyclonal patient IgG. (**a**) Outline of experimental set up. (**b**–**d**) Percentage of AChR clustering with a size larger than 15 µm^2^ induced by an agonist antibody or negative control antibody (Mota) with (+) or without (−) 13-3B5/b12 antibody (**b**), 11-3F6/b12 antibody (**c**) or MuSK MG polyclonal IgG (**d**) together with agrin. Data information: all data is normalized to the agrin only condition. Percentages of rescue are calculated with the following formula, taking the levels of negative controls in account: $\frac{\left(agrin+inhibition+agonist\right)-\left(agrin+inhibition\right)}{agrin only-untreated}\times {100}$. For statistical analysis, an unpaired t-test with Bonferroni correction was performed to compare the different agonists versus Mota control. Statistical significance was set at *P < 0.05; **P < 0.01; ***P < 0.001. #mAb13 experiments were performed with separate untreated and Mota conditions (not displayed), the 8 datapoints of mAb13 with and without agrin + Pt Total IgG were gathered from repetitions within two experiments instead of four individual experiments.

Exposure of myotubes to MuSK MG patient IgG4 can recapitulate pathological features of MuSK MG observed in mice injected with patient serum/IgG, such as AChR fragmentation and inhibition of MuSK phosphorylation^11,28,31,32^. To investigate the therapeutic potential of MuSK agonists on AChR clustering under myasthenic conditions in vitro, we exposed myotubes to agrin and antagonistic patient polyclonal antibodies or monoclonal monovalent MuSK antibodies (13-3B5/b12 and 11-3F6/b12). Thirty minutes later the agonistic bivalent monoclonal MuSK antibodies or a control antibody were added (Fig. 2a).

The minimal concentration for maximum inhibition (antagonist antibodies) or activation (agonist antibodies) of AChR clustering were determined using concentration-range-finding experiments (Supplementary Fig. S3). MuSK agonists 13-3D10a, 13-4D3a, 11-3F6c and 11-3D9b were able to (partially) oppose AChR declustering in vitro to levels of 35% to 130% compared to agrin-only induced AChR clusters (= 100%), depending on the clone and the antagonist used (Fig. 2b-d). Antibody 13-3D9b was the most potent in preventing/reversing pathogenic effects of polyclonal MuSK MG patient IgG or monovalent MuSK antibodies. The rescue of AChR cluster levels was ~ 1.5–2 fold larger in myotubes exposed to the monoclonal antagonists than when using polyclonal patient IgG for modelling MuSK MG. Interestingly, SIMPLE antibody 9E6, which (partially) binds to the same epitope as 11-3F6c, was able to induce clustering after inhibition by 13-3B5/b12, but unable to induce clustering after inhibition by 11-3F6/b12, suggesting that competition (with a higher affinity antibody) may be an important mechanism for agonist/antagonist function. Fz-binding antibody mAb13 was marginally able to rescue AChR clustering between 15 and 35% (Fig. 2b,c). Rescue of AChR clustering in these experiments occurred in all cluster sizes (Supplementary Fig. S4). In conclusion, MuSK agonist antibodies are able to (partially) rescue in vitro myasthenic effects in C2C12 myotubes.

### MuSK Ig-like 1 domain agonist antibodies do not improve MuSK myasthenia gravis in vivo

**Figure 3:**
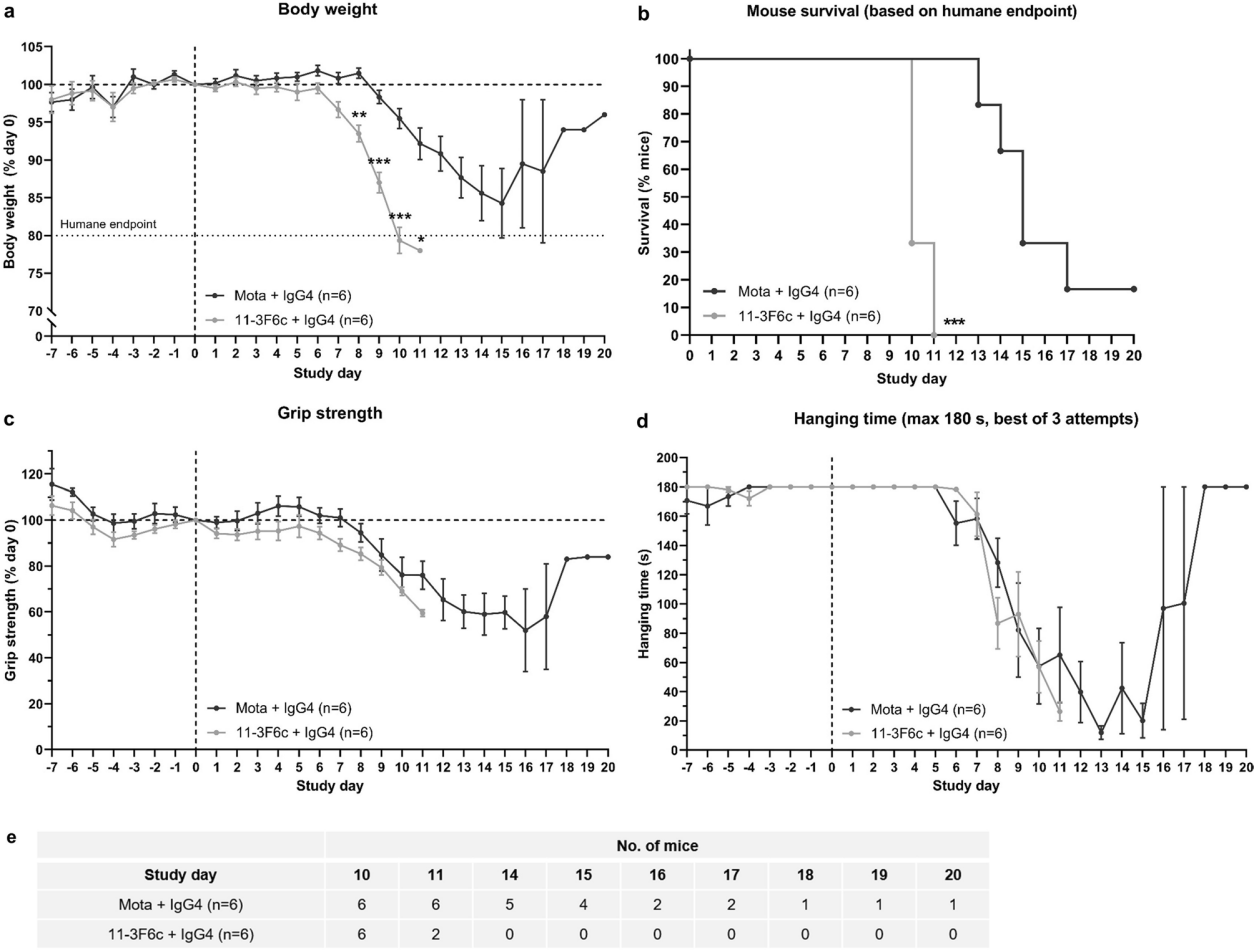
An Ig-like 1 binding MuSK agonist antibody does not improve myasthenic phenotype in a passive transfer MuSK MG (NOD/SCID) mouse model. (**a**) Percentage body weight of baseline (study day 0) values (n = 6/group). (**b**) Percentage survival of mice treated with prophylactic twice-weekly 2.5 mg/kg 11-3F6c or Mota. (**c**) Percentage grip strength of baseline (study day 0) values. (**d**) Hanging time on an inverted mesh (maximum 180 s, best of 3 attempts). (**e**) Number of (surviving) mice on the different study days included in the clinical outcome analyses. Data information: data are presented as mean % ± SEM (**a**,**c**) or mean ± SEM (**d**). For statistical analyses in (**a**), a multiple unpaired t-test with Holm–Šídák correction for multiple testing was performed to compare the 11-3F6c versus Mota group, and in (**b**), a Log-rank (Mantel–Cox test) was performed. Statistical significance was set at *P < 0.05; **P < 0.01; ***P < 0.001. Study design in Supplemental Fig. 1.

 Next, we investigated the therapeutic potential of one of the most potent agonists, 11-3F6c, in our MuSK MG IgG4 passive transfer model in NOD/SCID mice (^11^; study design in Supplementary Fig. S1e). Strikingly, 11-3F6c-treated mice had significantly greater body weight loss than mice treated with the negative control Mota antibody, and reached the humane endpoint of ≥ 20% body weight loss earlier than Mota-treated mice (Fig. 3a,b). However, no statistically significant differences were observed between the two treatment groups with regards to loss of grip strength (Fig. 3c) and hanging time in the inverted mesh test (Fig. 3d). It appears as though the Mota group recovered from day 17 onwards for all clinical outcomes, but this was due to one (remaining) Mota-treated mouse with a milder disease course surviving until the last day of the experiment (Fig. 3b,e). Overall, a MuSK Ig-like 1 domain agonist antibody therefore did not improve muscle weakness in the MuSK MG IgG4 passive transfer mouse model.

### MuSK Ig-like 1 agonist antibodies do not induce clinical MG but cause sudden death in wild type male C57BL/6 mice

**Figure 4:**
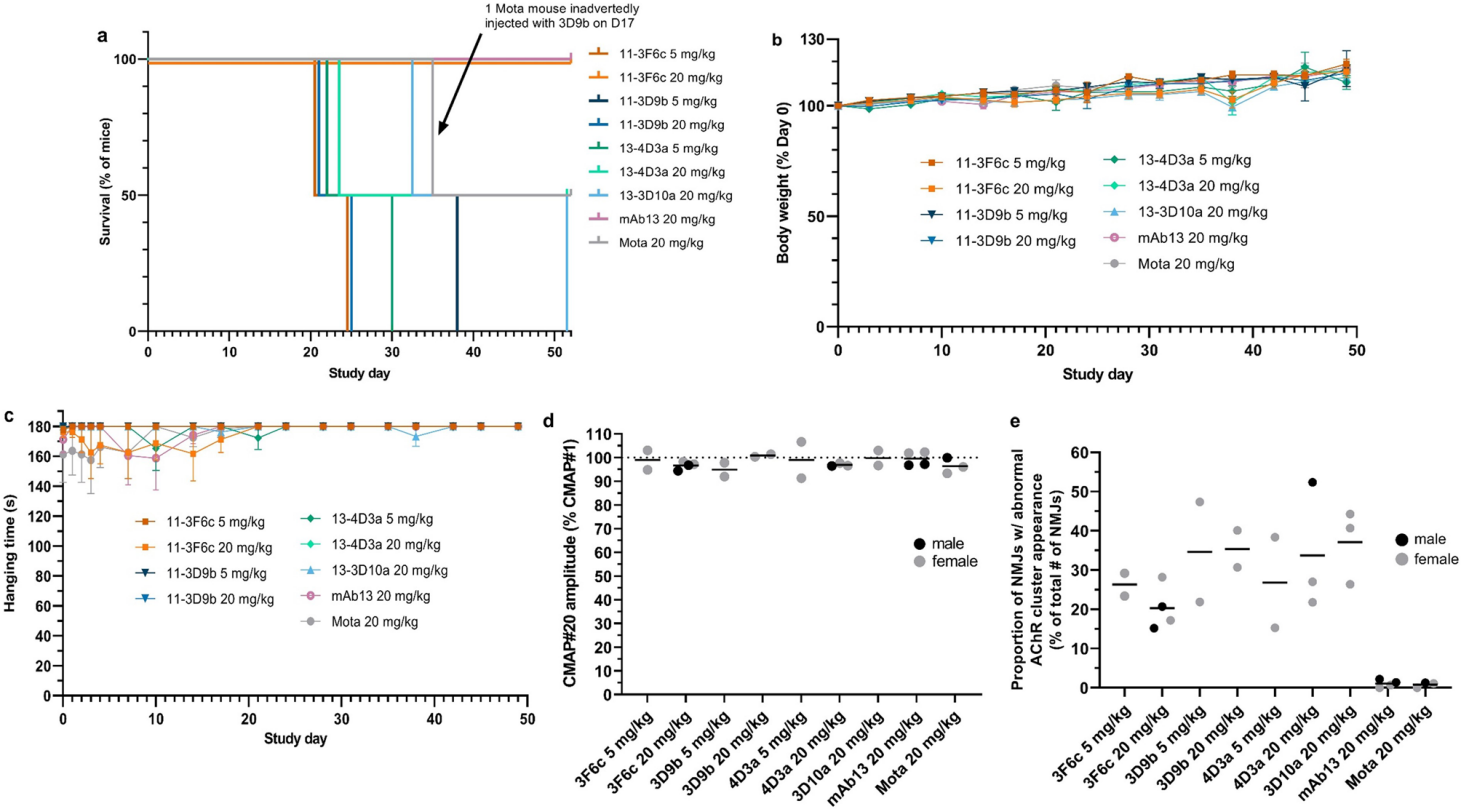
Ig-like 1 MuSK agonist antibodies cause sudden death in male C57BL/6 mice, but no (sub)clinical myasthenia. (**a**) Percentage survival of male C57BL/6 mice treated with MuSK agonist or control antibodies for 8 weeks (n = 2/group). All female mice (n = 2/group) survived. (**b**) Percentage body weight loss of baseline (study day 0, ±SEM) in all surviving mice (both male and female). (**c**) Hanging time (±SEM) in all surviving mice (both male and female). Graphed values represent the best attempt out of 3 (maximum 180 s). (**d**) Percentage of the average CMAP amplitude recorded after the 20th stimulus in the left leg of surviving mice at the end of the 8-week treatment. (**e**) The proportion of fragmented AChRs (percentage of total number of NMJs) in the diaphragm of all surviving mice at the end of the experiment.

To exclude the possibility that MuSK Ig-like 1 agonist antibodies induce MG on their own, we investigated the effect of long-term exposure to four MuSK agonists in wild-type mice (in the Besançon lab). Hereto, male and female (each n = 2) C57BL/6 mice were exposed to 5 or 20 mg/kg of MuSK agonist antibodies every 3 days for 8 weeks. Surprisingly, from 3 weeks of treatment onwards (range 21–52 days), the majority of male mice dosed with the MuSK Ig-like 1 agonist antibodies (10 of the 14 male mice) died without any preceding signs of reduced health or body weight loss (Fig. 4a). Necroscopy revealed enlarged bladders with urogenital abnormalities in at least 6 of the 9 carcasses examined (see below). One male mouse, dosed with 20 mg/kg 13-3D10a, had to be euthanized on day 51 of the experiment due to reaching the humane endpoint criteria. Upon pathological inspection it appeared to have an enlarged and hematomatous urinary bladder. All female mice dosed with MuSK agonist antibodies, as well as all male and female mice dosed with the Fz-binding MuSK agonist antibody mAb13 or Mota isotype control antibody remained unaffected and completed the experiment unremarkably. One male mouse in the Mota group erroneously received a 11-3D9b injection on day 17 and died with similar urogenital abnormalities on day 35. None of female mice and the surviving male mice exhibited any clinical myasthenic signs (Fig. 4b,c). In agreement, when in vivo NMJ function in calf muscles was assessed at the endpoint with RNS-EMG at 40 Hz stimulation of the sciatic nerve, no CMAP decrement was observed in surviving mice (Fig. 4d). However, when NMJ morphology of the diaphragm of the surviving male and female mice that had been dosed with the MuSK agonist antibodies was semi-quantitively assessed, it appeared that 15–52% of the NMJs had abnormal AChR area. Mice dosed with mAb13 and the negative control antibody Mota, displayed no abnormal NMJs (0–2.2% for both antibodies) (Fig. 4e).

### MuSK Ig-like 1 domain agonist antibodies cause mouse strain-specific and dose-dependent lethality in male mice

**Figure 5:**
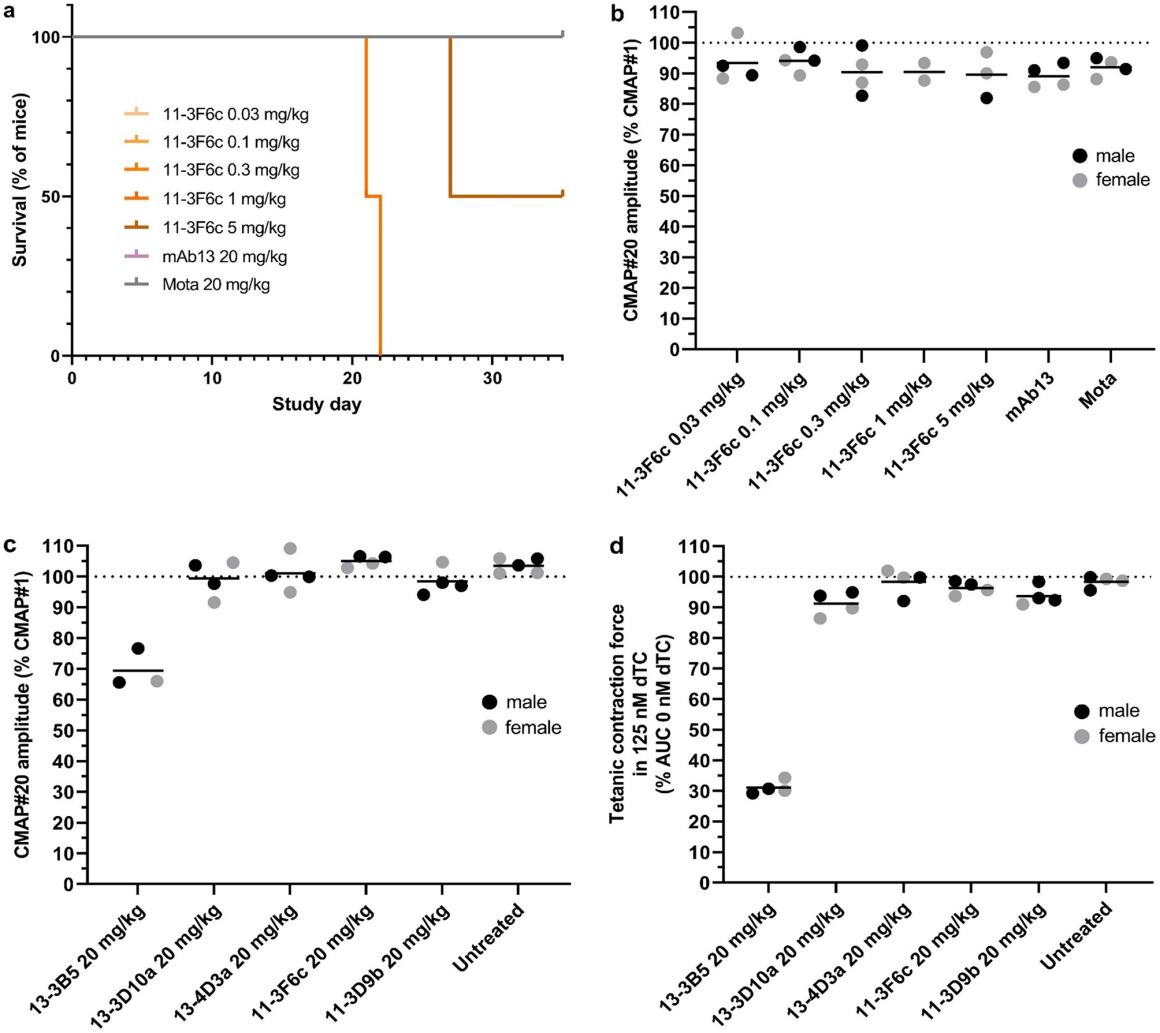
Ig-like 1 MuSK agonist candidate antibodies induce dose-dependent lethality but not myasthenia in C57BL/6 or NOD/SCID mice. (**a**) Percentage survival of male C57BL/6 mice treated with ascending doses of MuSK agonists or control antibodies for 5 weeks (n = 2/group). All female mice (n = 2/group) survived. (**b**) Amplitude of the 20th CMAP (expressed as percentage of the first), recorded in the left leg upon 40 Hz nerve stimulation of surviving C57BL/6 mice at the end of the 5-week treatment (n = 4/group). (**c**) Amplitude of the 20th CMAP (expressed as percentage of the first), recorded in the left leg upon 40 Hz nerve stimulation, of NOD/SCID mice at the end of 3.5-week treatment with 4 MuSK agonist antibodies, the pathogenic monospecific MuSK agonistic antibody 13-3B5 as a positive control and no treatment as negative control (n = 4/group, except for untreated group which had 3 males and 2 females). (**d**) Tetanic contraction force of left hemidiaphragm in all NOD/SCID mice at the end of the 3.5-week treatment with the MuSK agonists or control antibodies in 125 nM d-tubocurarine, normalized to the tetanic contraction force in 0 nM d-tubocurarine.

To investigate dose-dependency and mouse strain-specificity of the lethal effects, the study was repeated in wild type C57BL/6 (JAX laboratory) with lower doses and in NOD/SCID mice (LUMC). Of the four male C57BL/6 mice dosed with 1 or 5 mg/kg 11-3F6c every three days, three mice (two 1 mg/kg dosed, one 5 mg/kg dosed) suddenly died between days 20 and 30 (Fig. 5a) with a similar urogenital phenotype as observed before. No lethality was observed in the six male mice receiving lower doses (0.03, 0.1 and 0.3 mg/kg, every three days, n = 2 per dose) suggesting the effect (of possibly even one dose) is dose-dependent. All female mice receiving any of the dosing remained unaffected.

In NOD/SCID mice dosed twice per week with 20 mg/kg of the agonist antibodies for 3.5 weeks, no sudden death with urogenital pathology occurred in either male or female mice (data not shown). The lethal effect of MuSK Ig-like 1 agonist antibodies could therefore be strain specific, although additional mouse strains would have to be tested to confirm this. Again, no myasthenic phenotype or CMAP decrement was observed in surviving mice dosed with the candidate antibodies in either study (Fig. 5b,c). However, the contraction of diaphragms of 13-3D10a and 11-3D9b dosed NOD/SCID mice seemed slightly more sensitive to 125 nM d-tubocurarine than the untreated controls, but this was not significant (Fig. 5d). Altogether, these results suggest that the lethal action of the MuSK Ig-like 1 agonist antibodies is limited to the male C57BL/6 mice tested.

### The subclinical myasthenic features induced by MuSK Ig-like 1 domain agonist antibodies are non-progressive and possibly diaphragm-specific

To determine if the subclinical MG phenotype in mice exposed to the Ig-like 1 domain targeting agonists was progressive, we repeated the tolerability experiment in C57BL/6 mice for 11-3F6c, exposing male and female mice to 5 mg/kg every three days for 5, 8 or 11 weeks. In this study, 3 out of 9 male mice died or had to be sacrificed after 17, 24 and 73 days of treatment, respectively. They displayed the same urogenital pathology as described above (Supplementary Fig. S5a). Again, all female mice and control antibody-dosed mice remained unaffected. Serum antibody titres of the mice confirmed exposure to 11-3F6c had been comparable in all mice (Supplementary Fig. S5b). Furthermore, no CMAP decrement could again be observed in calf muscles of surviving mice at their respective endpoints (Supplementary Fig. S5c). However, there was a minor, non-progressive, statistically significant reduction in ex vivo diaphragm contraction force by 125 nM d-tubocurarine in mice treated for 8 and 11 weeks with 11-3F6c, compared with the Mota group (p < 0.01; Supplementary Fig. S5d). This indicates some loss of safety factor of neuromuscular transmission. In agreement, the diaphragm of (surviving) mice treated with 11-3F6c showed a proportion (~ 10%) of histologically abnormal NMJs, i.e. with fragmentation of AChRs, versus the Mota group at all time points (Supplementary Fig. S5e). However, the degree of fragmentation was not progressive. These subtle pathological effects of the agonist antibodies on the diaphragm might be due to higher initial exposure of the diaphragm to the intraperitoneally injected antibody as well as higher uptake of injected antibodies by the diaphragm versus the skeletal muscles^33^. To assess whether muscles that were only exposed indirectly (i.e. via the blood stream), the ETA, a forelimb muscle distant from the intraperitoneal injection site, was examined for histologically abnormal NMJs as well. However, no increased level of abnormal NMJs were observed in this muscle of 11-3F6c-treated mice, compared with controls (Supplementary Fig. S5e). This suggests that some degree of fragmentation of NMJ AChR area in diaphragm may be due to a disproportionally high exposure to the injected antibodies. This may have been caused by some degree of monovalent binding of the bivalent 11-3F6c antibody to MuSK, which has pathogenic effects^24,25^.

### Urogenital pathology in male C57BL/6J mice which died during MuSK Ig-like 1 domain agonist antibody treatment

**Figure 6:**
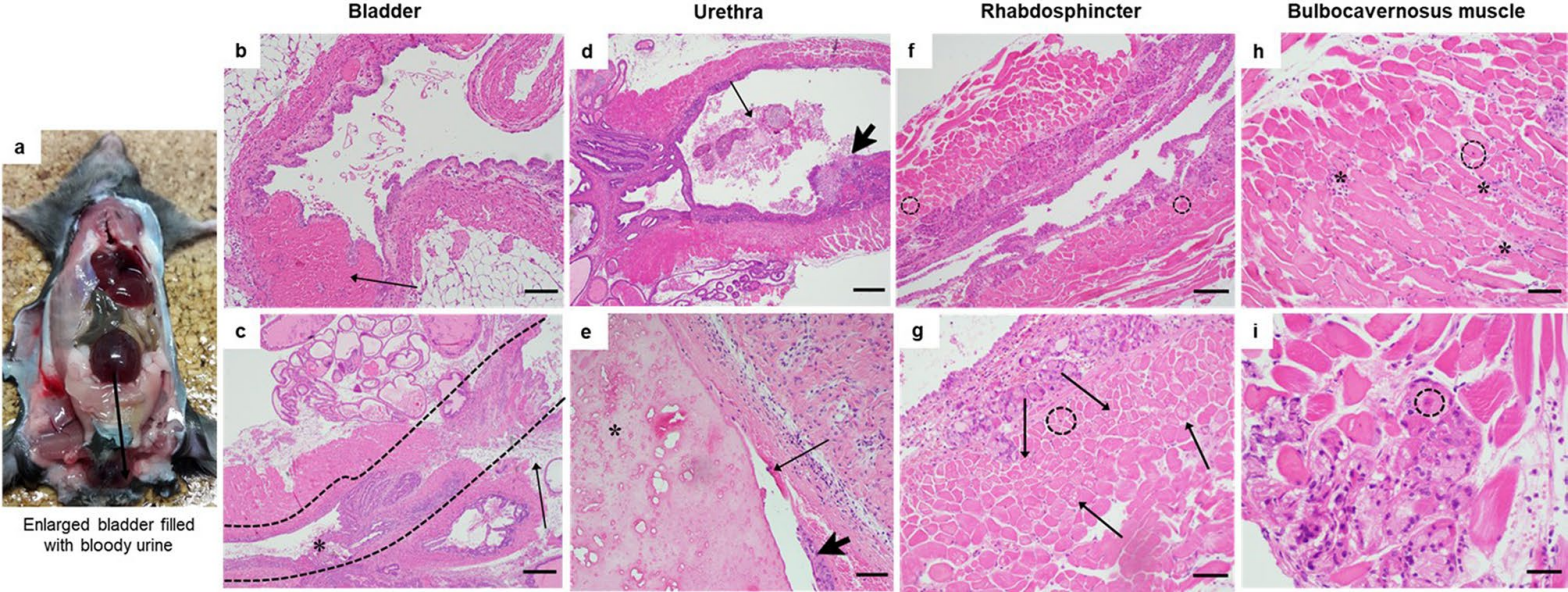
Histology of the urogenital system of male C57BL/6 mice showing pathology consistent with mouse urologic syndrome. (**a**) Example of a markedly distended bladder filled with (bloody) urine which was found in a male mouse that had died acutely. (**b**,**c**) In the bladder wall (**b**) and bladder neck/proximal urethra (**c**) hemorrhaging marked by extravascular erythrocytes and fibrin could be observed with some submucosal oedema and neutrophilic inflammation (arrow: dotted line indicates proximal urethra, left up to the trigone of the bladder, right). (**d**) The proximal penile urethra was filled with large amounts of proteinaceous material, erythrocytes and leukocytes (arrow). (**e**) In the distal urethra, there were moderate amounts of proteinaceous material (asterisk), marked mucosal ulceration and neutrophilic inflammation (arrow indicates area of ulcerated urethra, arrowhead indicates normally epithelialized region of urethra). (**f**,**g**) In the urethra at the level of the prostate, there was hyper-eosinophilia (**f**) and muscular degeneration in the rhabdosphincter (vacuolization: arrows, **g**) and occasional centralization of nuclei (dashed lines circles, **f**,**g**). (**h**,**i**) The pelvic musculature (bulbo-and ischio-cavernosus muscles) showed moderate inflammation (asterisks) and degeneration (dashed lines circle). Scale bar represents 25 µm.

To understand the pathology underlying the urogenital phenotype in male C57BL/6J mice, we dissected the urogenital system from the dead/euthanized male mice, and performed histopathological analyses. The macroscopic finding of a hematomatous, distended bladder filled with (bloody) urine (Fig. 6a) was also evident on a microscopic level by the presence of multifocal, moderate-to-severe haemorrhaging of the urinary bladder wall and extravascular erythrocytes and fibrin (Fig. 6b). The submucosa and musculature of the bladder wall were also characterized by moderate oedema and mild acute (neutrophilic) inflammation, which extended to the adjacent connective tissue until the level of the bladder neck/proximal urethra (Fig. 6c). The proximal and distal urethral lumen was multifocally dilated and contained moderate-to-large amounts of proteinaceous material and spermatozoa intermixed with erythrocytes and leukocytes, which was indicative of a urethral obstruction (Fig. 6d,e). In the distal urethra, there was also evidence of focal ulcerations and mild associated inflammation (Fig. 6e). The urethral inflammation extended into the musculature. The rhabdosphincter, a striated skeletal muscle surrounding the urethra that is involved in controlling the flow of urine from the bladder, showed hyper-eosinophilia and vacuolization of myocytes with occasional centralization of nuclei (Fig. 6f,g). Furthermore, there was macroscopic evidence of swelling of the pelvic musculature at the base of the penis i.e., the bulbocavernosus (or bulbospongiosus muscle (BCM), which plays a role in expulsion of urine and semen from the penis^34,35^. Histologically, the BCM was characterized by mild-to-moderate inflammation and degeneration of myocytes (Fig. 6h,i). Altogether, the cellular inflammation, degeneration and ulceration observed in the urogenital system of the male mice who died acutely, appeared to be secondary to the proteinaceous obstruction in the urethra, and is consistent with mouse urologic syndrome, a well-known condition in (older) male mice of several strains^36^.

### MuSK is localized at NMJs of the BCM and rhabdosphincter

The unexpected urogenital pathology in male C57BL/6 mice led us to hypothesize that MuSK Ig-like 1 agonist antibodies may bind and influence MuSK function at a structure within the urogenital system. So far, there is only evidence of MuSK gene expression in the bladder of the urogenital system in humans^37^. We therefore performed immunofluorescent stainings for MuSK using the MuSK agonist antibodies in wild type C57BL/6 mouse urogenital system and co-stained with fluorescently-labelled α-bungarotoxin to identify AChRs/NMJs.

Of the MuSK Ig-like 1 antibodies, 11-3F6c, 13-3D10a and 13-3B5wt clearly stained the BCM (Supplementary Fig. S6a) and rhabdosphincter (Supplementary Fig. S6b) of wild-type C57BL/6 mice. The staining co-localized with AChRs, indicating that these antibodies bind to MuSK at the NMJs of these tissues. Antibodies 11-3D9b and 13-4D3a, but not 11-3F6c, 13-3D10a and 13-3B5wt, stained the bladder wall (Supplementary Fig. S6c). Furthermore, the Fz-binding antibodies, mAb13 and 1E11, broadly stained the rhabdosphincter and BCM, but none of these stainings co-localized with AChRs. The same broad staining was observed in the bladder wall, suggesting these antibodies may bind aspecifically to these tissues. The staining patterns observed in the urogenital tissues were similar for the prostate gland (Supplementary Fig. S6c), as the epithelial layers in this structure were (markedly) stained by 11-3D9b, 13-4D3a, mAb13 (although no urogenital pathology was observed with mAb13) and 1E11 (the toxicity of this antibody on male was not tested), and only marginally or not at all by 11-3F6c, 13-3B5wt and 13-3D10a. Altogether, the data indicate that the various antibodies bind differentially to structures within the urogenital system, with only 11-3F6c and 13-3D10a evidently staining for MuSK at NMJs in the rhabdosphincter and BCM. Since broad non-NMJ specific staining of these structures was also observed with mAb13, which did not induce a phenotype, it remains unclear whether (a)specific binding is responsible for the pathological phenotype and what is the underlying mechanism.

## Discussion

**Figure 7:**
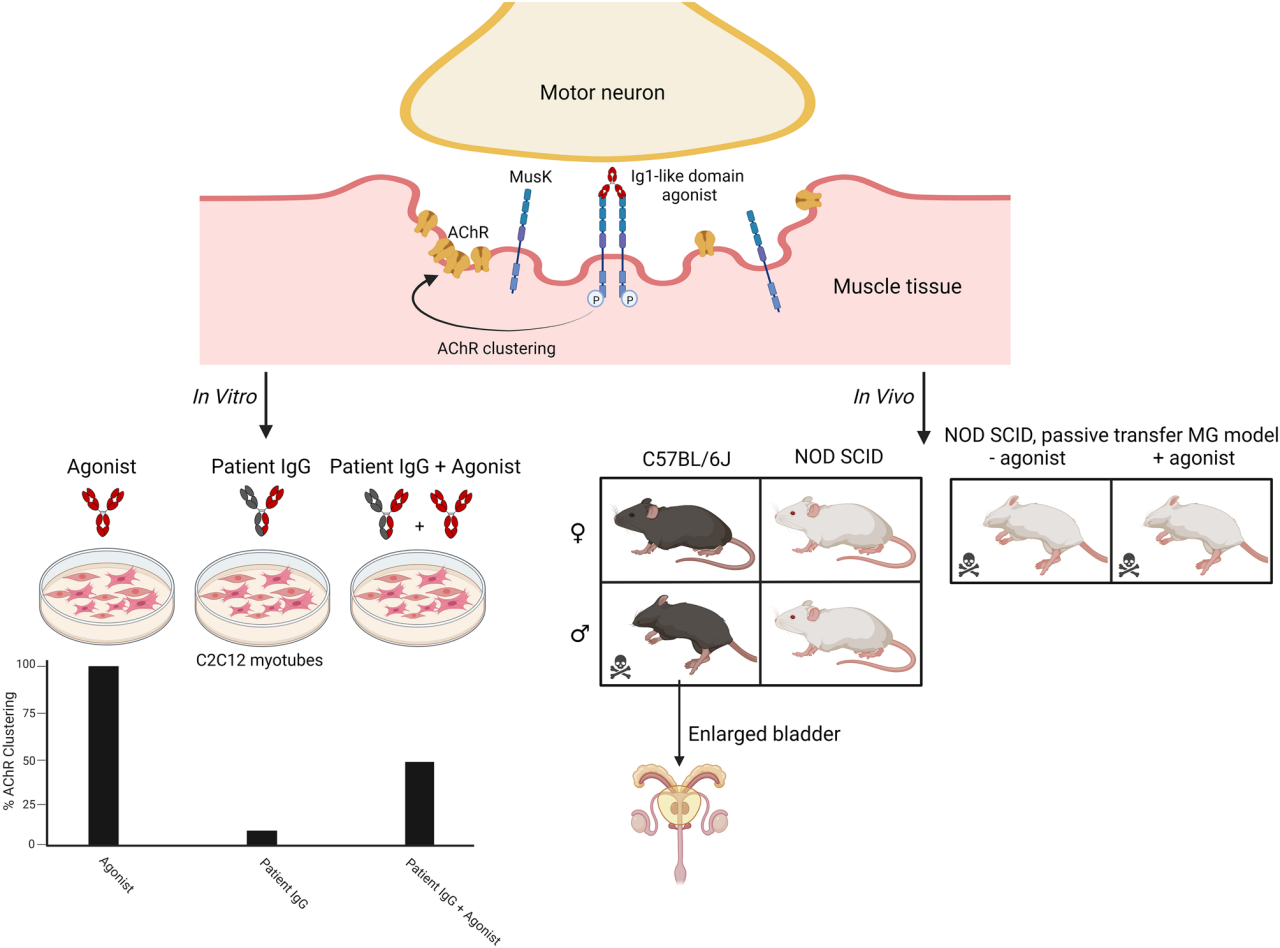
Graphical overview of main conclusions. Agonist antibodies binding to the Ig-like 1 domain of MuSK at the postsynaptic muscle membrane, induce MuSK phosphorylation and AChR clustering to different degrees. In vitro these agonist antibodies are able to partially rescue inhibition of this cascade induced by MuSK MG patient IgG. In vivo injection of agonist MuSK antibodies resulted in death in a large proportion of male C57BL/6 mice, due to an enlarged bladder/urogenital syndrome. Female mice and NOD/SCID mice did not suffer from enlarged bladders nor lethality. MuSK Ig-like 1 domain agonist antibodies were also unable to rescue progressive MuSK MG in a passive transfer NOD/SCID mouse model. Figure was created with http://BioRender.com.

Therapeutic targeting of the agrin-Lrp4-MuSK-Dok7 trophic signalling cascade may be a promising strategy for treatment of NMDs characterized by NMJ impairment^1,2^. Here, we explored the potential of MuSK Ig-like 1 domain binding monoclonal agonist antibodies to rescue AChR de-clustering and NMJ instability. While in vitro (therapeutic) agonistic potential of these antibodies was shown, in the passive transfer mouse model one of these antibodies was tested but found not capable of rescuing the myasthenic phenotype. Furthermore, our in vivo mouse studies uncovered an as yet unknown action of MuSK Ig-like 1 domain binding agonists on the urogenital system, which caused mouse strain- and male-specific lethal pathology in a proportion of mice (Fig. 7).

We generated, modified and characterized 25 different monoclonal bivalent antibodies targeting the MuSK Ig-like 1 domain. The antibodies activated MuSK phosphorylation and AChR clustering in C2C12 myotubes to a varying degree. Interestingly, full activation of the MuSK kinase did not necessarily result in full activation of AChR clustering. In addition, Ig-like 1 domain MuSK agonist antibodies produced smaller sized (i.e. less “mature”) clusters than agrin. Thus, the manner by which MuSK is dimerized and activated appears to be critical for subsequent signalling and appropriate cluster formation. Our data suggests that Ig-like 1 domain MuSK antibodies may have differential effects from agrin, such as: (1) alter the sequence or timing of phosphorylation of MuSK tyrosines, making it less effective or capable of recruiting downstream factors like Dok7^38,39^, (2) change the configuration of the MuSK dimer making it less capable of downstream signalling, or (3) obstruct interaction with other proteins such as Lrp4. These results suggest that artificial MuSK agonism is possible, but entails additional unknown requirements to exactly mimic the effects of the natural signalling molecule agrin. Interestingly, co-incubation of the antibodies with agrin did not have a synergistic effect, but resulted in AChR cluster counts equal to using the agonist antibody only. This suggests competition of binding with agrin-Lrp4 to MuSK or a conformational change of MuSK preventing binding of agrin-Lrp4 could play a role herein. The MuSK Fz domain binder mAb13 also does not induce more clusters when co-incubated with agrin. In fact, similar to the Ig-like 1 binding antibodies, mAb13 induced more smaller clusters, but less mature clusters than agrin. The lack of synergy with a Fz-binder may be partly due to the fact that Lrp4 can actually bind both the Ig-like 1 and Fz domain of MuSK^40^, thereby also giving some competition for binding. mAb13 and agrin however did elicit similar downstream phosphorylation signalling cascades in C2C12 cells^41^. Altogether, this suggests the forced MuSK dimerization by the antibodies overrules binding of agrin-Lrp4 to MuSK and that more factors are involved in successful AChR cluster formation than phosphorylation of MuSK alone.

When considering the in vitro myasthenic model in C2C12 myotubes, it is clear that MuSK agonism can bypass the inhibitory effect of patients antibodies through at least two modes of action: (1) competition for binding as evident from the negative correlation between affinity and AChR clustering levels and rescue experiments using clones from similar and different epitopes, and (2) forced dimerization, based on partial rescue observed using bivalent clones with different epitope specificities. Based on these cellular experiments, it was surprising to not observe any beneficial effect of the MuSK agonist 11-3F6c in our MuSK MG IgG4 passive transfer model. In the long-term C57BL/6J tolerability study, we observed minor but significant AChR fragmentation induced by 11-3F6c in diaphragm NMJs. This may have also occurred in the MuSK MG animals, contributing to the observed accelerated body weight loss. The lack of morphological abnormalities in NMJs of limb muscle of the C57BL/6J mice suggests that the intraperitoneal route of administration may disproportionally affect the diaphragm in this mouse model by creating a temporary locally high dose of MuSK antibodies resulting in pathogenic monovalent binding. This matches previous MuSK MG mouse model experiments where therapeutic treatment with efgartigimod, an FcRn inhibitor, clearly improved limb muscle function, but showed only subtle improvements of diaphragm NMJ function^42^. Future preclinical experiments in MG animal models may therefore benefit from administration routes other than intraperitoneal. Another possibility is that due to competition of binding between 11-3F6c and agrin-Lrp4 to MuSK, and the already lower expression of MuSK in the MuSK MG mouse model particularly in the bulbar muscles^43,44^, critical levels of MuSK are reached more quickly resulting in accelerated weakness compared to e.g. the limb muscles.

This study furthermore uncovered an unexpected role for MuSK in the urogenital system in mice. All four candidate Ig-like 1 domain agonist antibodies tested in vivo caused unexpected urogenital pathology, most likely responsible for the observed sudden death in the male C57BL/6 mice. This resembled mouse urologic syndrome, a well-known condition, in which normal male mice are found dead with no prior signs of illness, or as a chronic form in which mice can live for up to 12 months^36,45^. In our experiments, a proportion of male mice died suddenly at variable treatment duration periods, starting only after three weeks of injections. For reasons still unclear, one injection of a (high-dose) MuSK Ig-like 1 antibody was sufficient to induce this sudden death. Thus far, urogenital pathology has never been reported in either active immunization or passive transfer models for MuSK MG in mice, rats or rabbits^11,30,46^. Importantly, the mouse urogenital system is not identical to that of humans; urethral plugs and the observed urologic syndrome are also not inherent to humans. This therefore does not rule out that the Ig-like 1 antibodies could have no urogenital side-effects in humans. Furthermore, urogenital structural or functional abnormalities are also not known to be a symptom in (MuSK) MG patients^47–49^. A recent prospective questionnaire-based study reported that AChR MG patients (no MuSK MG patients were included) had more often an overactive bladder than healthy controls, possibly explained by the use of pyridostigmine^50^. MuSK MG patient autoantibodies predominantly target the same Ig-like 1 domain of MuSK as our patient-derived clones^51^, as well as the antibodies used in our animal models. This suggests that the urogenital pathological phenotype may be specific for and limited to the (C57BL/6) male mice tested.

Furthermore, a fully functioning (adaptive) immune system may be important as immuno-incompetent NOD/SCID mice did not display the urogenital pathology. However, the monoclonal antibodies used in our models contain mutations limiting their inflammatory and binding capacity with immune cells or complement, suggesting that immunocompetency of the animal model could be irrelevant. The mechanism underlying the observed strain differences therefore remains not understood.

The presence of MuSK and cholinergic neurons and AChRs in the bladder and/or skeletal muscles controlling bladder voiding^52,53^ suggests binding of the Ig-like 1 agonistic antibodies to MuSK in the urogenital system plays a role in this pathological phenotype in mice. The urogenital system is mostly operated by smooth muscles although some sphincters (rhabdosphincter) and supporting skeletal muscles are closely associated in the pelvic floor (bulbocavernosus muscle). One hypothesis could be that MuSK agonism, through an unknown mechanism, prevents such skeletal muscle from completing ejaculation in male mice. When semen is not fully removed from the urogenital tract, such material might sporadically cause obstruction by enlarging the urethral plugs that are an endogenous feature in healthy male mice, produced for copulation^54,55^. This may underlie the urogenital pathological symptoms observed in our mice. Alternatively, the MuSK Ig-like 1 agonistic antibodies may have impacted the urogenital system through another as yet unidentified route. Importantly, either route appears to be dependent on the binding of MuSK agonist antibodies to the Ig-like 1 domain of MuSK, as the Fz-binder mAb13 did not induce urogenital abnormalities. This may be due to the presence of MuSK splice variants or differences in MuSK protein in the urogenital system as a result of glycosylation or other post-translational modifications. This in turn could result in an absent or masked Fz domain thereby simply preventing the binding of the MuSK Fz domain agonists to MuSK. Indeed, our immunohistochemical stainings could not confirm specific MuSK staining in NMJs of the rhabdosphincter and BCM using the Fz domain binding antibody.

In light of recent publications^17,21,23^, therapies that enhance MuSK activation remain a promising avenue to explore as a treatment for MuSK MG as well as other NMDs, such as SMA and ALS. In this study we developed and characterised a gamma of different agonist antibodies binding the MuSK Ig-like 1 domain. In vitro, a clear beneficial effect of agonist antibodies was found, but in MuSK MG mice a therapeutic benefit of this panel of antibodies was not found. Moreover, the sudden death in a large proportion of male C57BL/6 mice call for caution in further development of these antibodies for therapeutic use, but does not rule out that other Ig-like 1 domain antibodies binding different epitopes could be promising therapeutics. This study has advanced our understanding on how to achieve successful MuSK agonism and shows that MuSK activation is a critical process that cannot be successfully mediated by all MuSK binding bivalent antibodies. Instead, epitope and mechanism of activation of the MuSK kinase are important factors. Future research should aim to further understand these processes and to investigate whether other (Ig-like 1 domain) antibodies do hold therapeutic promise, thereby facilitating preclinical development of MuSK agonists toward treatment for NMDs.

## Supplementary Information

Supplementary Information.

## Data Availability

The datasets used and/or analysed during the current study are available from the corresponding author on reasonable request.
