# Supplementary material for: Development and characterization of agonistic antibodies targeting the Ig-like 1 domain of MuSK
Source: Sci Rep. 2023 May 8;13:7478. doi: 10.1038/s41598-023-32641-1 (PMC10167245; doi:10.1038/s41598-023-32641-1)
Supplement: Supplementary file 1 — Supplementary Information. [file 41598_2023_32641_MOESM1_ESM.pdf]

SUPPLEMENTARY APPENDIX

Development and characterization of agonistic antibodies targeting the Ig-like 1 domain of MuSK

Jamie L. Lim* PhD[1], Roy Augustinus* MSc[1], Jaap J. Plomp PhD[2], Kasra Roya-Kouchaki MSc[1], Dana L.E. Vergoossen MSc[1], Yvonne Fillié-Grijpma[1], Josephine Struijk BSc[1], Rachel Thomas DVM[3], Daniela Salvatori DVM, PhD, Dipl.ECVP [4], Christophe Steyaert MSc[5], Christophe Blanchetot PhD[5], Roeland Vanhauwaert PhD[5], Karen Silence PhD[5], Silvère M. van der Maarel PhD[1], Jan J. Verschuuren MD, PhD[2], Maartje G. Huijbers PhD[1,2]**

*Contributed equally.
**Corresponding author.

Supplementary Table S1. Overview of antibody characteristics of the parental clones and their germlined humanized variants.

Antibody clone	Origin	Epitope	ka (1/Ms)		kd (1/s)		KD (nM)	
			Mouse MuSK	Human MuSK	Mouse MuSK	Human MuSK	Mouse MuSK	Human MuSK
13-3B5wt	MuSK MG Patient	Ig1	4.43E+05	2.01E+05	1.97E-05*	3.70E-05	0.04*	0.18
13-3D10wt	MuSK MG Patient	Ig1	1.91E+05	9.98E+05	1.73E-04	9.77E-05	0.91	0.01
13-3D10a	MuSK MG Patient	Ig1	7.33E+05	8.88E+05	7.28E-05	1.37E-04	0.10	0.15
13-4D3wt	MuSK MG Patient	Ig1	1.60E+05	6.74E+05	4.11E-05	9.64E-05	0.26	0.14
13-4D3a	MuSK MG Patient	Ig1	4.95E+05	6.11E+05	8.20E-05	1.57E-04	0.17	0.26
11-3F6wt	MuSK MG Patient	Ig1	5.56E+05	7.58E+05	7.29E-07*	2.08E-05	0.0013*	0.03
11-3F6c	MuSK MG Patient	Ig1	6.49E+05	6.41E+05	2.13E-05	3.19E-05	0.03	0.05
11-3D9wt	MuSK MG Patient	Ig1	8.89E+05	8.16E+05	9.82E-08	3.04E-05	0.00011*	0.04
11-3D9b	MuSK MG Patient	Ig1	3.89E+05	4.22E+05	5.55E-05	7.32E-05	0.14	0.17
9E6	llama	Ig1	2.88E+05	4.99E+05	6.80E-04	3.73E-04	2.36	0.75
1E11	llama	Fz	6.82E+05	7.40E+05	9.50E-06	1.08E-03	0.01	1.47
mAb13	Recombinant production based on Xie (1997)	Fz	4.8E+05	No binding	6.7E-04	No binding	1.4	No binding

*Values that are nearing the detection limit of the Biacore T200 system and therefore should be considered estimations of extremely low KD values.
ka, association rate constant; kd, dissociation rate constant; KD, equilibrium dissociation constant.

Supplementary Table S2. Overview of *in vivo* study designs and outcomes.

Study	C57BL/6 mice Besançon lab	C57BL/6 mice Jackson lab	NOD/SCID mice LUMC	C57BL/6J mice LUMC
**Dosing**	5 or 20 mg/kg of all MuSK Ig-like 1 domain agonist antibodies or control antibodies	0.03, 0.1, 0.3, 1 and 5 mg/kg 11-3F6c or control antibodies	20 mg/kg of all candidate agonist or control antibodies	5 mg/kg 11-3F6c or control antibodies
**Treatment duration**	8 weeks	5 weeks	3 weeks	5, 8, 11 weeks (11-3F6c) or 12 weeks (Mota)
**N per dosing group; total N**	4 mice (2 males and 2 females); 36 mice	4 mice (2 males and 2 females); 20 mice	4 mice (2 males and 2 females, 2 males and 3 females in untreated group); 25 mice	6 mice (3 males and 3 females); 24 mice
**Number of dead mice**	12/18 dead or humane endpoint-killed male mice with all antibodies and doses between Days 21 and 51. All females completed study.	3/4 male mice died with 1 mg/kg and 5 mg/kg between Days 20 and 30. All females completed study.	All males and females completed study	3/9 dead or humane endpoint-killed male 3F6c-treated mice died; all females completed study
**Pathological abnormalities**	Dead mice had enlarged bladders and urogenital abnormalities	Dead mice had enlarged bladders and urogenital abnormalities	No urogenital abnormalities	Dead mice had enlarged bladders and urogenital abnormalities
**Myasthenic phenotype**	No	No	No	No
**NMJ morphology**	~15-52% abnormal NMJs with all lead antibodies in diaphragm of surviving mice	Max. 10% abnormal NMJs with 5 mg/kg 11-3F6c (data not shown)	Max. ~10% abnormal NMJs with all lead antibodies in diaphragm of mice at end of experiment (data not shown)	~4-26% abnormal NMJs with 5 mg/kg 11-3F6c in diaphragm of surviving mice
**RNS-EMG (CMAP decrement)**	Not assessed	Not assessed	No CMAP decrement observed in mice at end of experiment	No CMAP decrement observed in surviving mice
**Diaphragm contraction (safety factor assessment. i.e. curare sensitivity)**	Not assessed	Not assessed	No changes in curare sensitivity observed	Minor (up to 2%) increase in curare sensitivity in surviving mice treated for 8 or 11 weeks with 11-3F6c, compared with control antibody

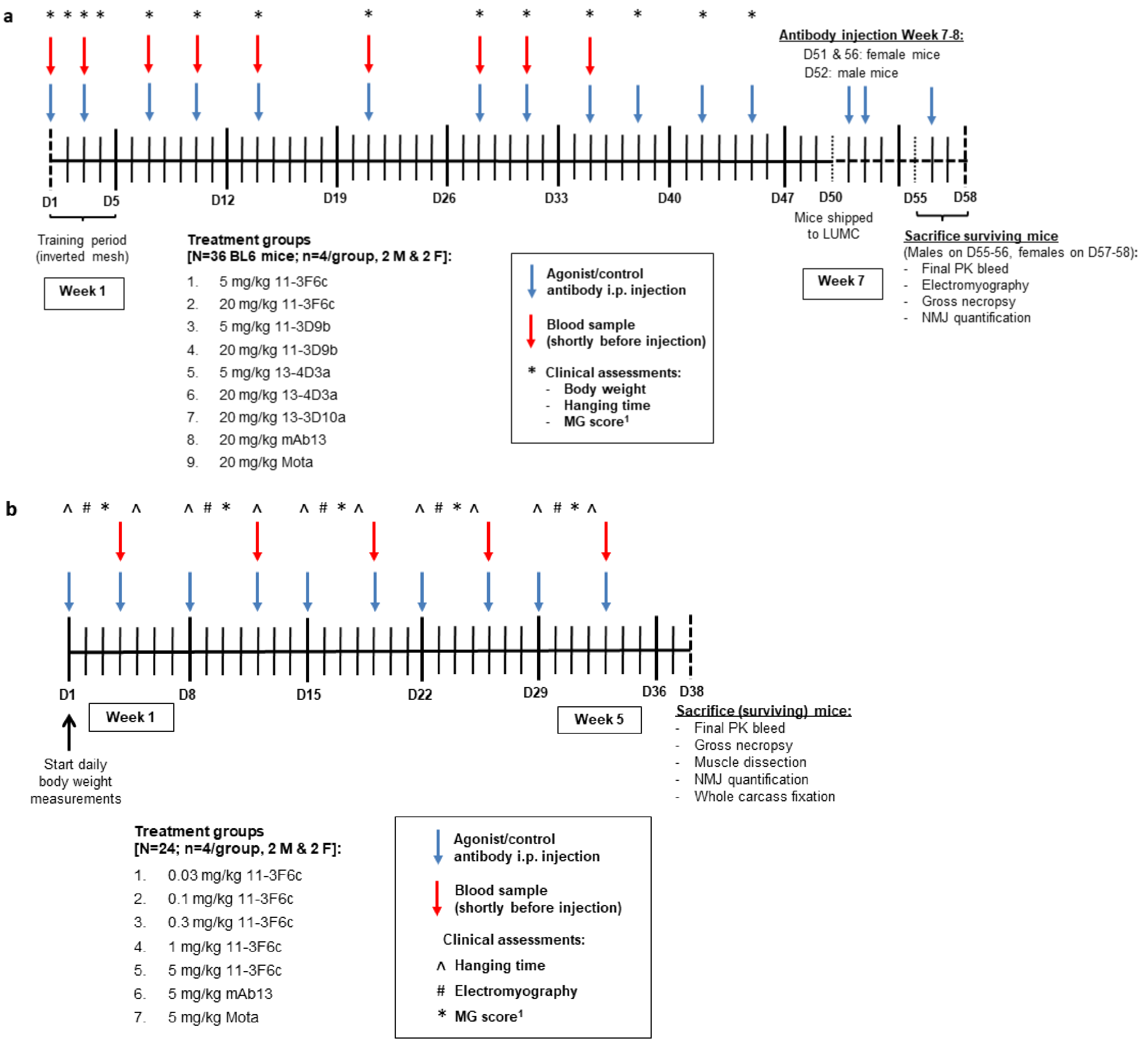
a
Antibody injection Week 7-8:
D51 & 56: female mice
D52: male mice
D1
D5
D12
D19
D26
D33
D40
D47
D50
D55
D58
Training period (inverted mesh)
Week 1
Mice shipped to LUMC
Week 7
Sacrifice surviving mice
(Males on D55-56, females on D57-58):
- Final PK bleed
- Electromyography
- Gross necropsy
- NMJ quantification
Treatment groups
[N=36 BL6 mice; n=4/group, 2 M & 2 F]:
1. 5 mg/kg 11-3F6c
2. 20 mg/kg 11-3F6c
3. 5 mg/kg 11-3D9b
4. 20 mg/kg 11-3D9b
5. 5 mg/kg 13-4D3a
6. 20 mg/kg 13-4D3a
7. 20 mg/kg 13-3D10a
8. 20 mg/kg mAb13
9. 20 mg/kg Mota
Agonist/control antibody i.p. injection
Blood sample (shortly before injection)
* Clinical assessments:
- Body weight
- Hanging time
- MG score1
b
D1
D8
D15
D22
D29
D36
D38
Week 1
Week 5
Start daily body weight measurements
Sacrifice (surviving) mice:
- Final PK bleed
- Gross necropsy
- Muscle dissection
- NMJ quantification
- Whole carcass fixation
Treatment groups
[N=24; n=4/group, 2 M & 2 F]:
1. 0.03 mg/kg 11-3F6c
2. 0.1 mg/kg 11-3F6c
3. 0.3 mg/kg 11-3F6c
4. 1 mg/kg 11-3F6c
5. 5 mg/kg 11-3F6c
6. 5 mg/kg mAb13
7. 5 mg/kg Mota
Agonist/control antibody i.p. injection
Blood sample (shortly before injection)
Clinical assessments:
ʌ Hanging time
Electromyography
* MG score1

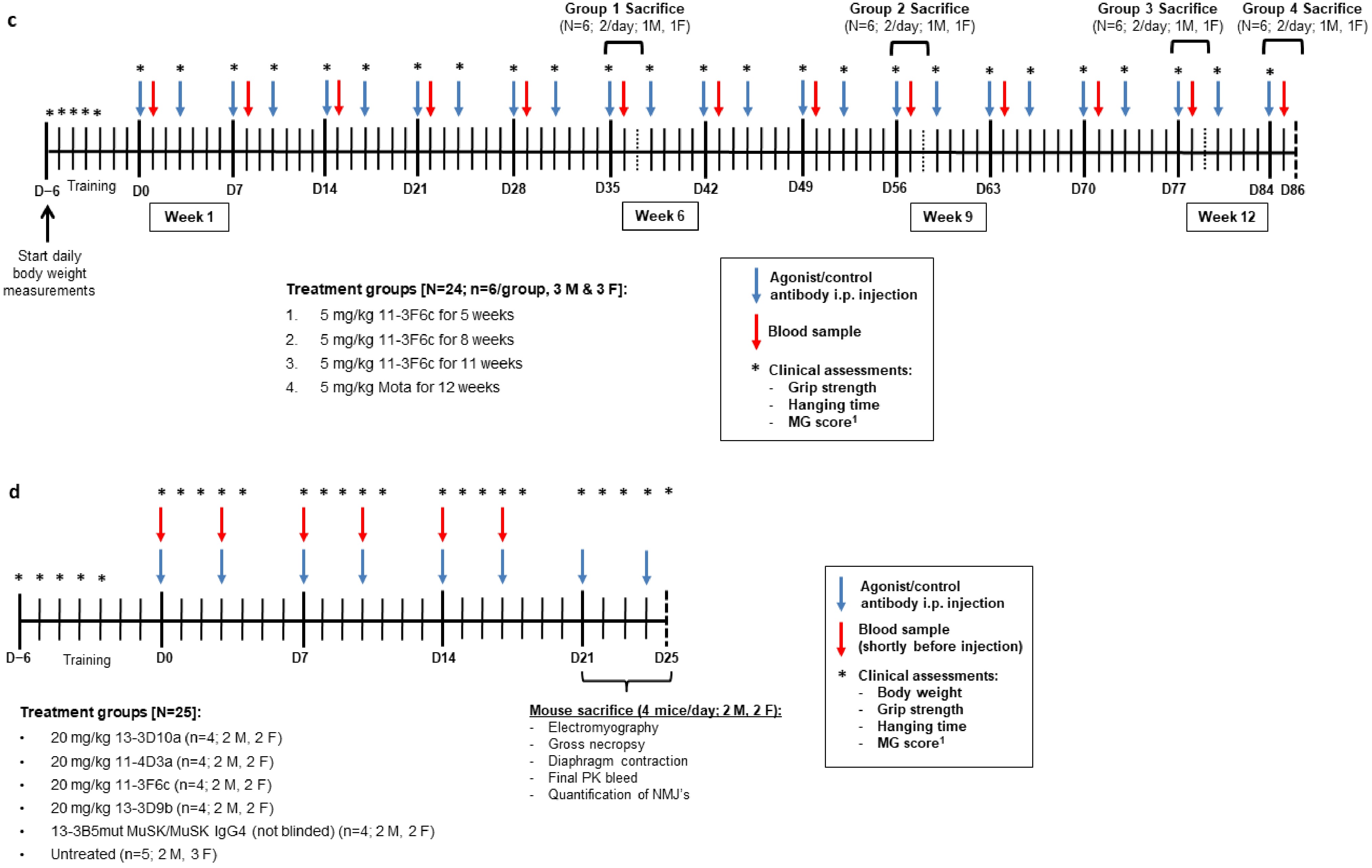
c
Group 1 Sacrifice
(N=6; 2/day; 1M, 1F)
Group 2 Sacrifice
(N=6; 2/day; 1M, 1F)
Group 3 Sacrifice
(N=6; 2/day; 1M, 1F)
Group 4 Sacrifice
(N=6; 2/day; 1M, 1F)
D−6
Training
D0
D7
D14
D21
D28
D35
D42
D49
D56
D63
D70
D77
D84
D86
Week 1
Week 6
Week 9
Week 12
Start daily body weight measurements
Treatment groups [N=24; n=6/group, 3 M & 3 F]:
1. 5 mg/kg 11-3F6c for 5 weeks
2. 5 mg/kg 11-3F6c for 8 weeks
3. 5 mg/kg 11-3F6c for 11 weeks
4. 5 mg/kg Mota for 12 weeks
Agonist/control antibody i.p. injection
Blood sample
* Clinical assessments:
- Grip strength
- Hanging time
- MG score1
d
D−6
Training
D0
D7
D14
D21
D25
Treatment groups [N=25]:
• 20 mg/kg 13-3D10a (n=4; 2 M, 2 F)
• 20 mg/kg 11-4D3a (n=4; 2 M, 2 F)
• 20 mg/kg 11-3F6c (n=4; 2 M, 2 F)
• 20 mg/kg 13-3D9b (n=4; 2 M, 2 F)
• 13-3B5mut MuSK/MuSK IgG4 (not blinded) (n=4; 2 M, 2 F)
• Untreated (n=5; 2 M, 3 F)
Mouse sacrifice (4 mice/day; 2 M, 2 F):
- Electromyography
- Gross necropsy
- Diaphragm contraction
- Final PK bleed
- Quantification of NMJ's
Agonist/control antibody i.p. injection
Blood sample (shortly before injection)
* Clinical assessments:
- Body weight
- Grip strength
- Hanging time
- MG score1

e

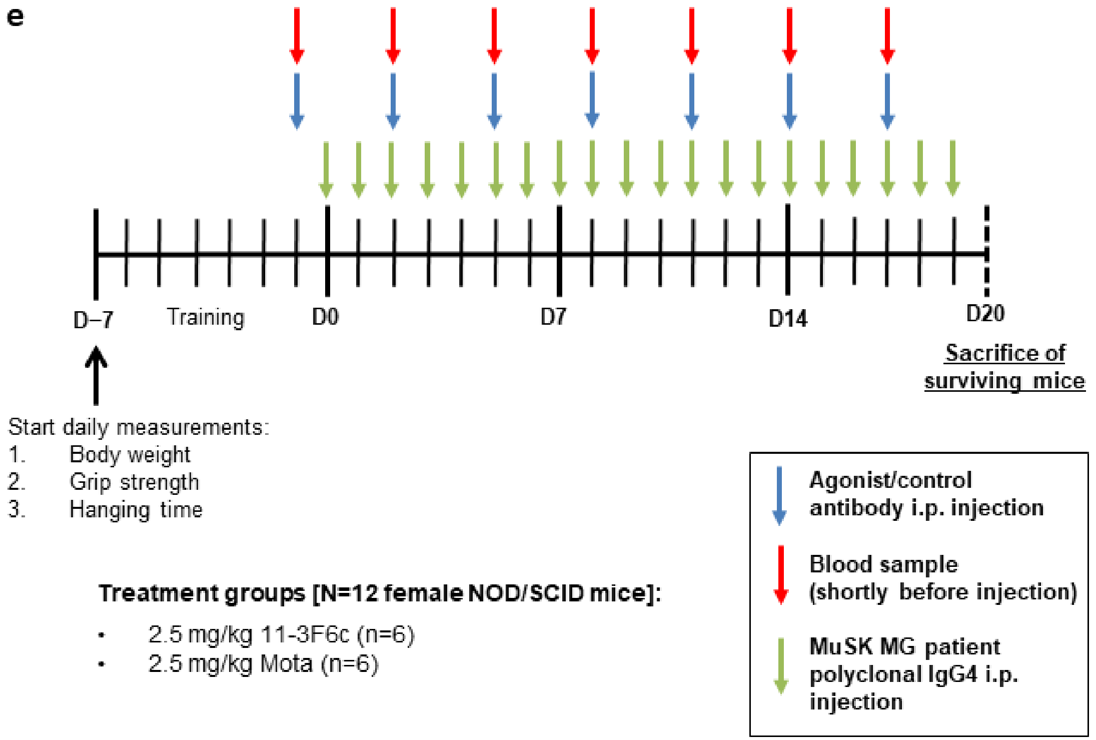

Supplementary Figure S1

Study designs of passive transfer tolerability experiments with Ig-like 1 agonist or control antibodies in C57BL/6 mice at the (**a**) Besançon laboratory, (**b**) Jackson laboratory and (**c**) LUMC. Study designs of the (**d**) tolerability study and (**e**) passive transfer MuSK MG experiment with patient polyclonal IgG4 and Ig-like 1 agonist or control antibody in NOD/SCID mice.

[1]MG score refers to weakness in mice that was visually scored as per Stacy et al., 2002 (0 = no weakness, 1 = weakness upon activity, 2 = weakness at rest, 3 = severe weakness with breathing difficulty, 4 = death). D, day; EMG, electromyography; F, female; MG, myasthenia gravis; M, male; PK, pharmacokinetics.

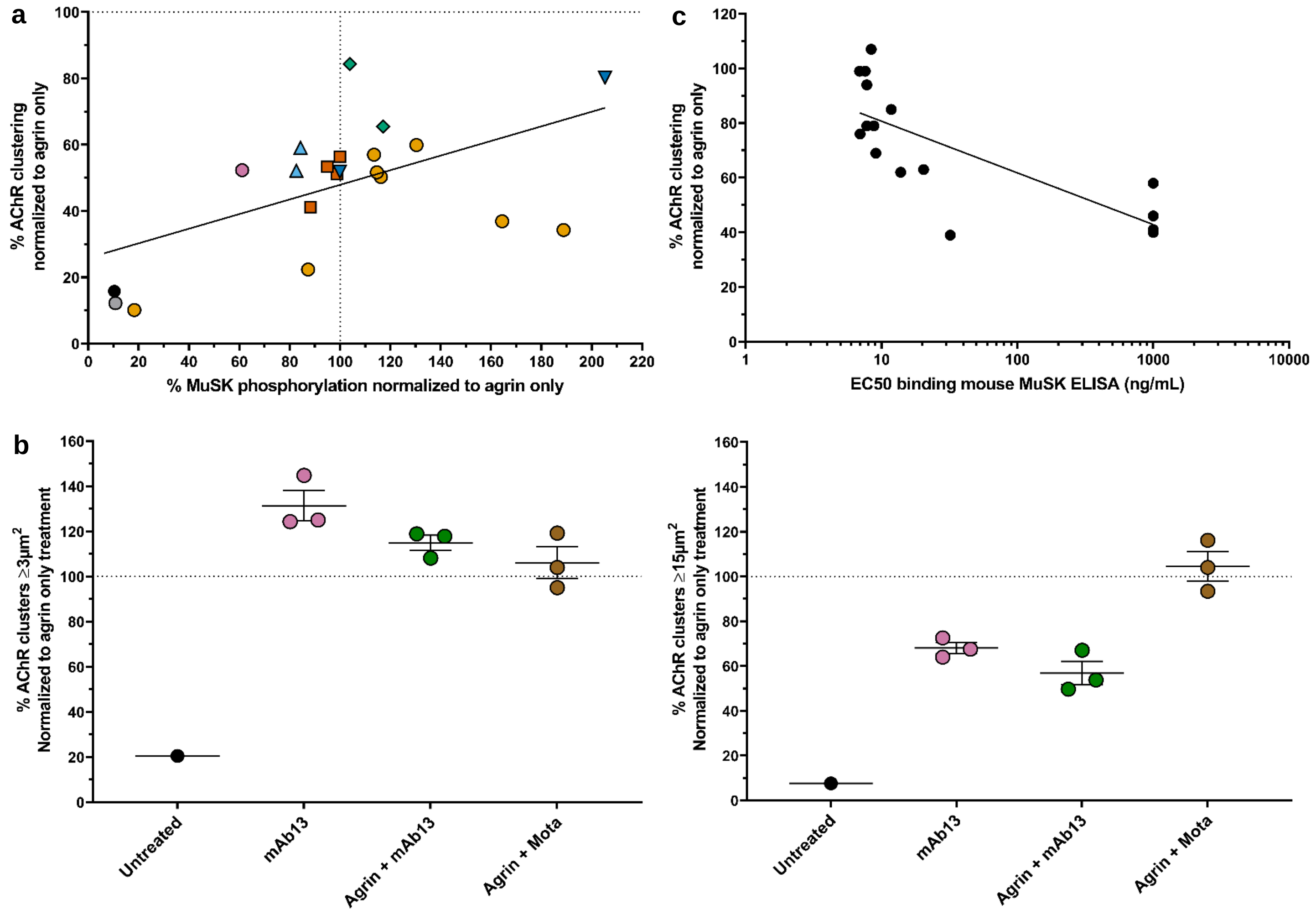

Supplementary Figure S2

a AChR clustering and MuSK phosphorylation both normalized to agrin show a significant positive correlation (R=0.59, P=0.0029 with a slope of 0.22). Colors identify groups of antibody clones derived from the same parental clone (dark yellow = 11-3B5, light blue = 13-3D10, dark blue = 11-3D9, dark orange = 11-3F6, light green = 4D3, pink = mAb13, grey = anti-biotin control, black = untreated).

b Percentage of AChR clustering normalized to agrin with a size ≥3 m^2 (left) and ≥15 m^2 (right) induced by untreated condition, negative control antibody (Mota) or agonist antibody mAb13 with or without agrin.

c AChR clustering normalized to agrin and EC50 binding affinity of different Ig-like 1 domain and Fz-domain MuSK agonist antibodies show a significant negative correlation ($R=-0.66$, $P=0.0049$ with a slope of -0.034).

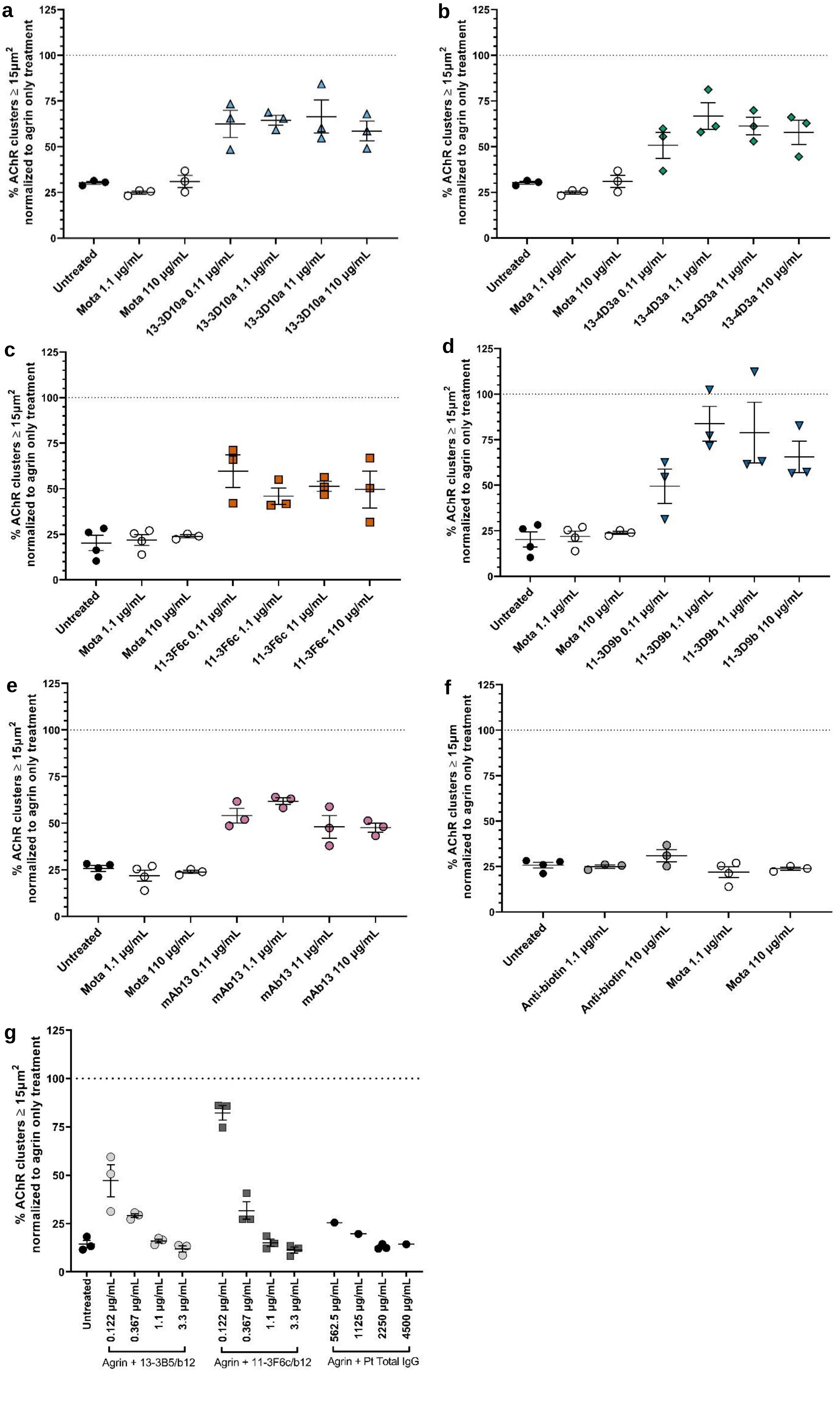
a
b
c
d
e
f
g
% AChR clusters ≥ 15μm²
normalized to agrin only treatment
% AChR clusters ≥ 15μm
125
100
75
50
25
0
Untreated
Mota 1.1 μg/mL
Mota 110 μg/mL
13-3D10a 0.11 μg/mL
13-3D10a 1.1 μg/mL
13-3D10a 11 μg/mL
13-3D10a 110 μg/mL
13-4D3a 0.11 μg/mL
13-4D3a 1.1 μg/mL
13-4D3a 11 μg/mL
13-4D3a 110 μg/mL
11-3F6c 0.11 μg/mL
11-3F6c 1.1 μg/mL
11-3F6c 11 μg/mL
11-3F6c 110 μg/mL
11-3D9b 0.11 μg/mL
11-3D9b 1.1 μg/mL
11-3D9b 11 μg/mL
11-3D9b 110 μg/mL
mAb13 0.11 μg/mL
mAb13 1.1 μg/mL
mAb13 11 μg/mL
mAb13 110 μg/mL
Anti-biotin 1.1 μg/mL
Anti-biotin 110 μg/mL
0.122 μg/mL
0.367 μg/mL
1.1 μg/mL
3.3 μg/mL
562.5 μg/mL
1125 μg/mL
2250 μg/mL
4500 μg/mL
Agrin + 13-3B5/b12
Agrin + 11-3F6c/b12
Agrin + Pt Total IgG

Supplementary Figure S3

a-g Dose response experiments for all candidate, control and antagonist antibodies. Based on these experiments 1.1 ug/mL was determined to be the minimal dose for maximal agonistic and minimal pathogenic effects.

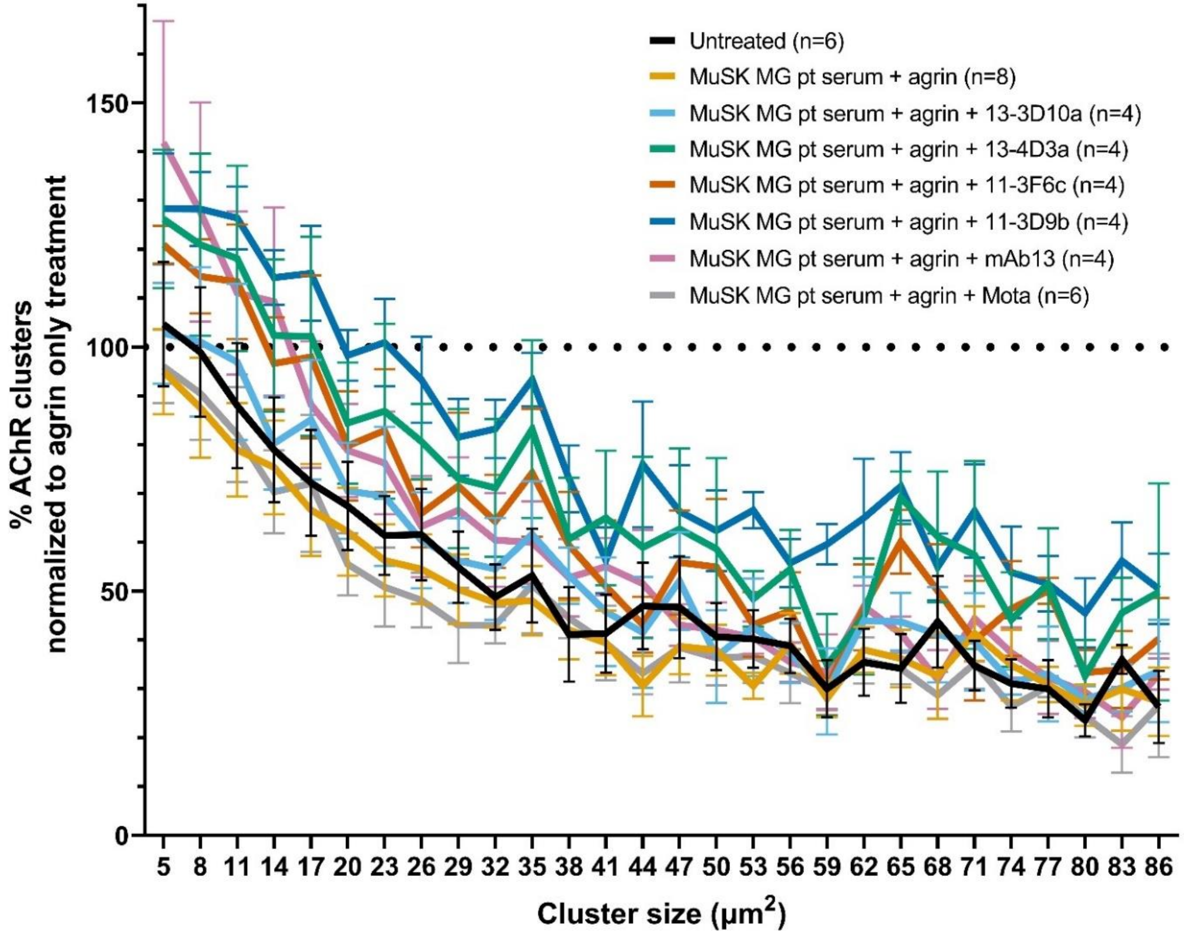

Supplementary Figure S4

Distribution of AChR cluster sizes for the different candidate antibodies in combination with MuSK MG patient serum, normalized to the agrin only condition.

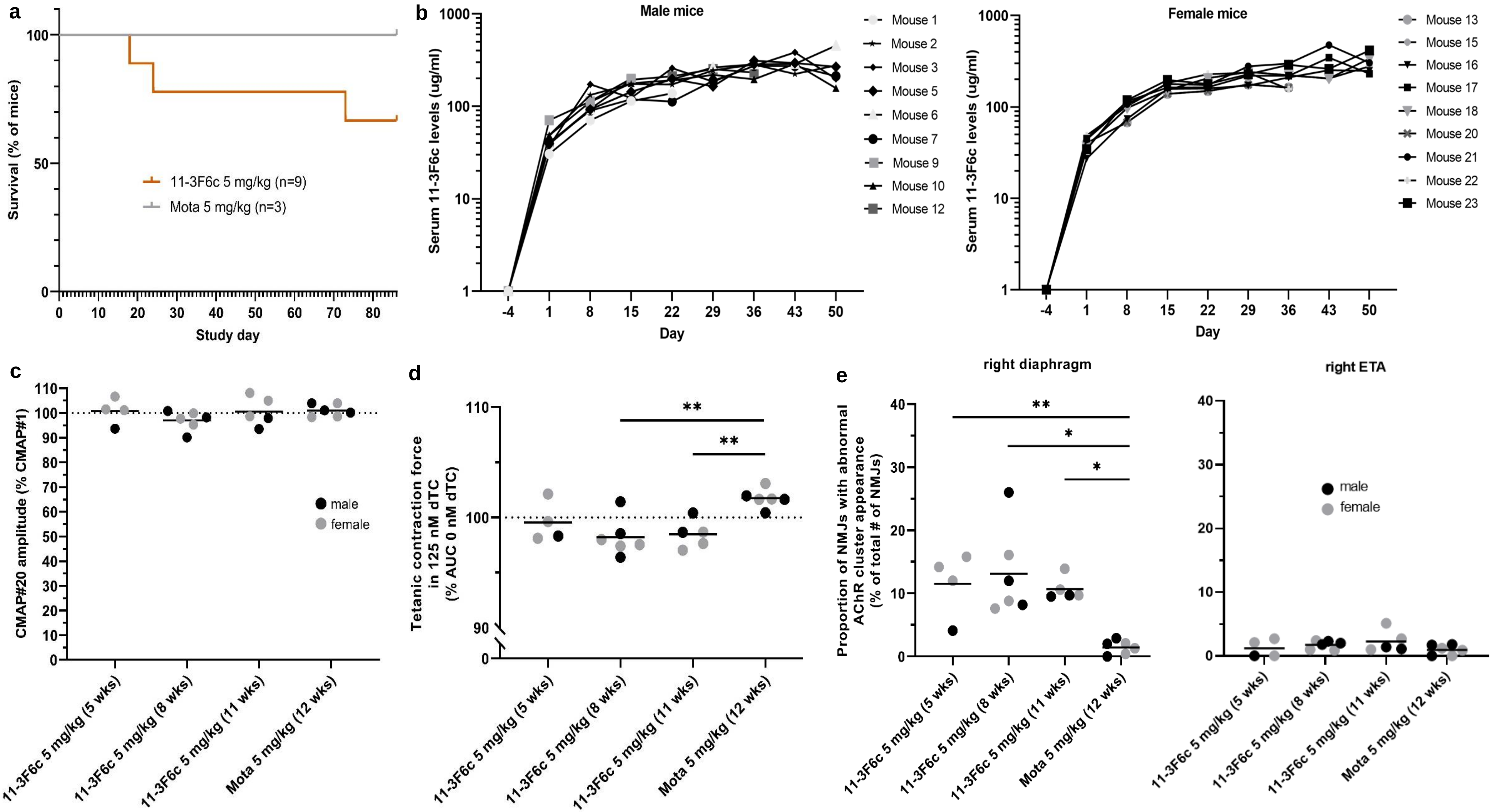
a
Survival (% of mice)
Study day
11-3F6c 5 mg/kg (n=9)
Mota 5 mg/kg (n=3)
b
Male mice
Female mice
Serum 11-3F6c levels (ug/ml)
Day
Mouse 1
Mouse 2
Mouse 3
Mouse 5
Mouse 6
Mouse 7
Mouse 9
Mouse 10
Mouse 12
Mouse 13
Mouse 15
Mouse 16
Mouse 17
Mouse 18
Mouse 20
Mouse 21
Mouse 22
Mouse 23
c
CMAP#20 amplitude (% CMAP#1)
male
female
d
Tetanic contraction force in 125 nM dTC (% AUC 0 nM dTC)
e
right diaphragm
right ETA
Proportion of NMJs with abnormal AChR cluster appearance (% of total # of NMJs)
11-3F6c 5 mg/kg (5 wks)
11-3F6c 5 mg/kg (8 wks)
11-3F6c 5 mg/kg (11 wks)
Mota 5 mg/kg (12 wks)

Supplementary Figure S5. An Ig-like 1 MuSK agonist antibody does not induce overt myasthenia in C57BL/6 mice.

a Percentage survival of male C57BL/6 mice treated with 5 mg/kg 11-3F6c or control antibody for up to 12 weeks (n=3/group). All female mice survived (n=3/group).

b Concentration of 11-3F6c agonist antibody in serum of C57BL/6 mice.

c Percentage of the average CMAP amplitude recorded after the 20th stimulus in the left leg of surviving C57BL/6 mice at endpoint (n=6/group).

d Tetanic contraction force of left hemidiaphragm in all surviving C57BL/6 mice at endpoint, recorded in the presence of 250 nM d-tubocurarine normalized to the tetanic contraction force in 0 nM d-tubocurarine.

e The percentage of fragmented AChRs in the right hemi-diaphragm (dorsal strip) of all surviving C57BL/6 mice at endpoint.

Data information: For statistical analysis in d and e, one-way-ANOVA with Dunnett's multiple comparison test was performed to compare the 11-3F6c- versus Mota-treated groups. Statistical significance was set at **P<0.01.

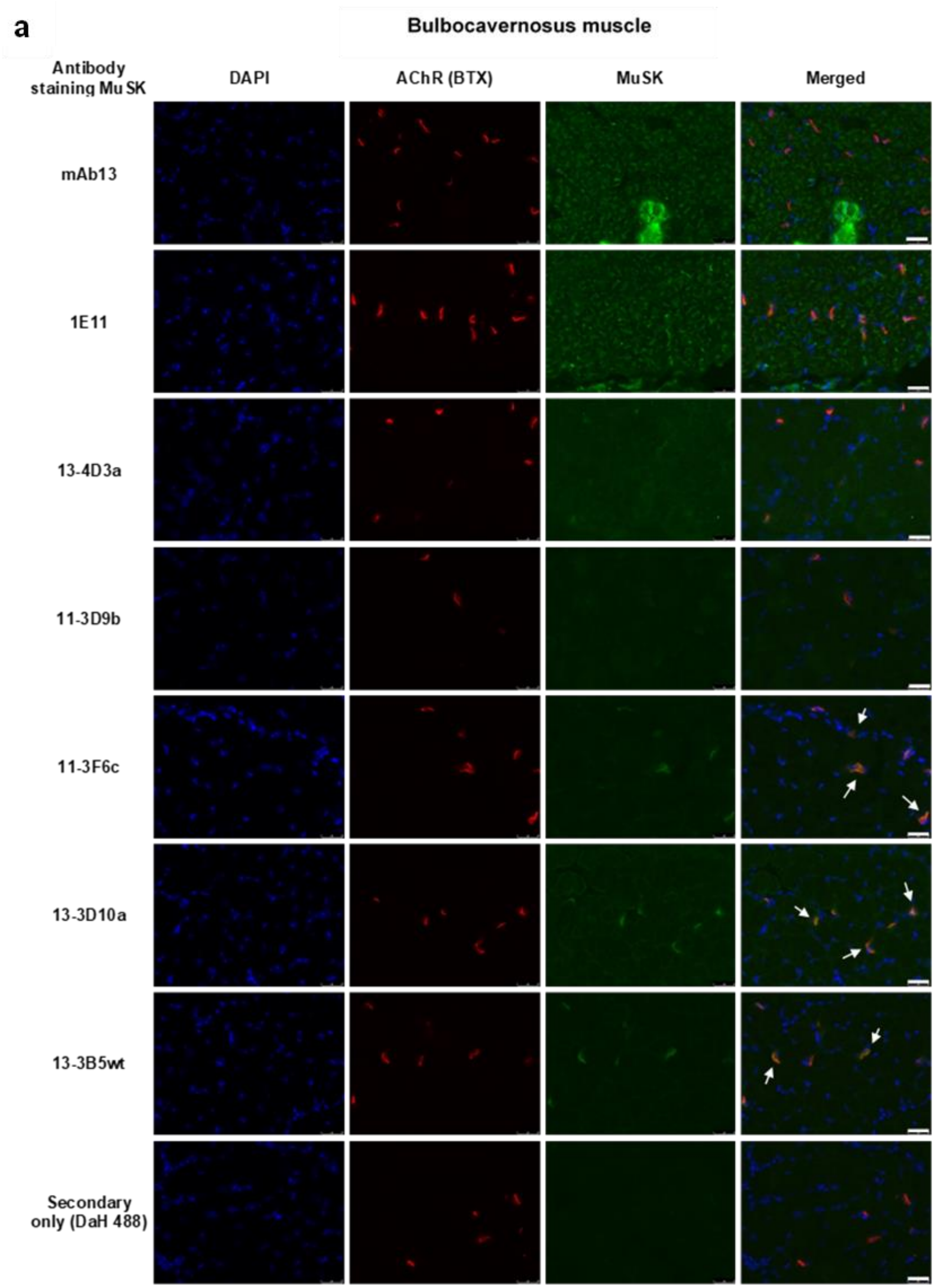
a
Bulbocavernosus muscle
Antibody staining MuSK
DAPI
AChR (BTX)
MuSK
Merged
mAb13
1E11
13-4D3a
11-3D9b
11-3F6c
13-3D10a
13-3B5wt
Secondary only (DaH 488)

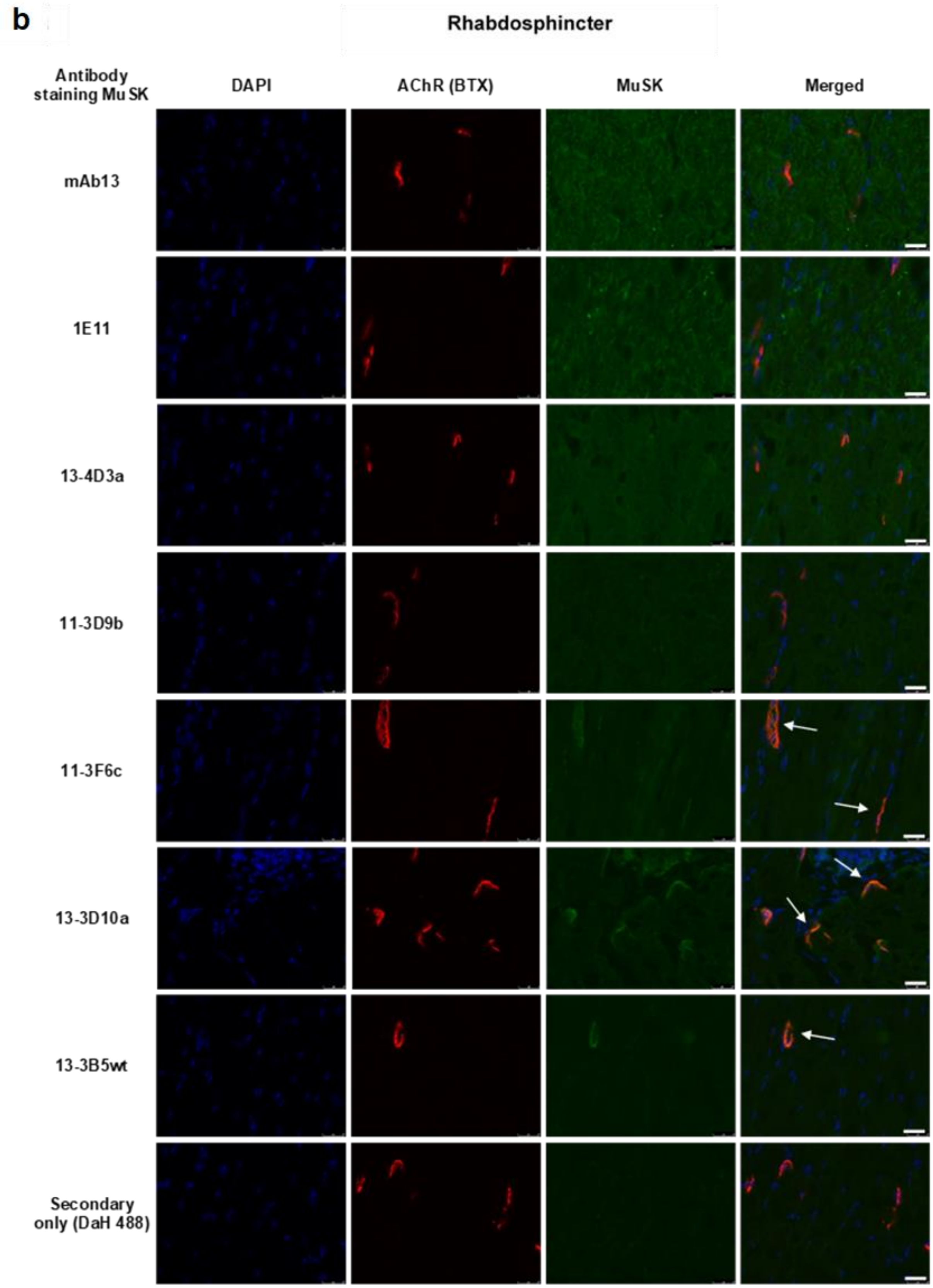

b
Rhabdosphincter
Antibody staining MuSK
DAPI
AChR (BTX)
MuSK
Merged
mAb13
1E11
13-4D3a
11-3D9b
11-3F6c
13-3D10a
13-3B5wt
Secondary only (DaH 488)

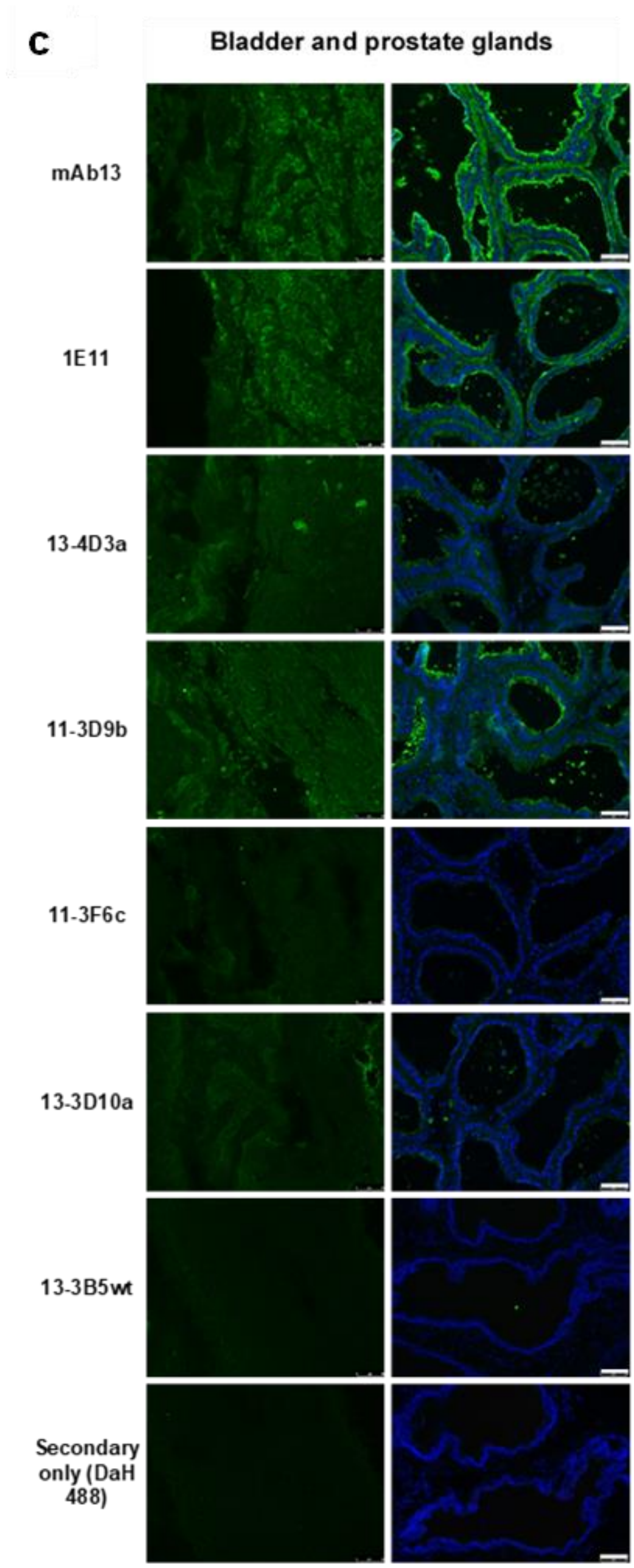

Supplementary Figure S6. Immunofluorescent stainings of wildtype C57BL/6 male mouse urogenital systems.

Immunofluorescent stainings of the (**a**) bulbocavernosus muscle, (**b**) rhabdosphincter, (**c**) bladder (left) and prostate glands (right) of a wildtype C57BL/6 male mouse. Blue color is DAPI staining of nuclei, red denotes alpha-bungarotoxin (BTX) marking AChRs/NMJs, and green represents MuSK agonist antibody staining. White arrows denote examples of MuSK-expressing NMJs.
Scale bar represents 50 µm.
